# Supplementary material for: Association of macro-level determinants with adolescent overweight and suicidal ideation with planning: A cross-sectional study of 21 Latin American and Caribbean Countries
Source: PLoS Med. 2020 Dec 29;17(12):e1003443. doi: 10.1371/journal.pmed.1003443 (PMC7771665; doi:10.1371/journal.pmed.1003443)
Supplement: S1 Analysis Plan — (DOCX) [file pmed.1003443.s001.docx]

**Statistical analysis plan**

**Study title:** "Association of macro level determinants with adolescent overweight and suicidal ideation with planning: a cross-sectional study of 21 Latin American and Caribbean Countries"

1. **Hypothesis**

- Within Low- and Middle-Income Countries in the Latin America and Caribbean region overweight/obesity will be associated with suicide ideation with planning. These associations will vary by sex.
- Social and economic inequalities in the LAC region have increased considerably over the last few decades as region’s economies transitioned from low to higher levels of economic development; we hypothesise that the likelihood of suicide ideation with planning or overweight/obesity association will be higher in countries with higher levels of national income, human development or income inequality. These associations will vary by sex.

1. **Objectives**

To examine (1) the association between national indices (economic growth , human development, and income inequality) and overweight/obesity, and suicidal ideation and planning in teenage boys and girls and (2) the association between overweight/obesity and suicidal ideation with planning in teenage boys and girls, independent of individual level risk factors

**3. Data source**

**Subjects and study design**

The study used the most recent Global School-based student Health surveys (GSHS) data from national representative samples of adolescents in 20 Latin American and Caribbean countries between 2009 and 2013. Argentina was not included initially because of the its large sample size and the authors assumed it would bias the results.

**🡪 Modifications carried out in the revised version**

In response to reviewers’ comments we added Argentina to the dataset and 21 countries were included in the final dataset since meta-analytical type methods are designed to deal with different sample sizes.

**Outcomes**

The GSHS dataset contains derived variables for underweight and overweight/obesity using international age and sex specific child BMI cut off points (>-2 SD and >+1 SD from the from median for age and sex based on WHO growth reference curves, respectively). We focused on overweight and obesity the number of underweight participants was too small to allow robust estimates.

A binary variable was derived to measure suicide ideation with planning (‘yes’ if an affirmative response to both ideation and planning; otherwise classified as ‘no’)

**Exposures**

Initially country and year-specific indices of Gross Domestic Product (GDP/capita) and the Human development Index (HDI) and Gini were extracted from the World Bank Group and the UNDP data and merged into the dataset. Gini was excluded due to missing data for over half of the LAC countries. GDP and HDI were then recorded into tertiles for ease of interpretation and to achieve greater statistical power, with the high GDP or HDI tertile being the reference category.

**🡪 Modifications carried out in the revised version**

Based on reviewers’ suggestions, we used the World Inequality Income Database which includes high quality data from the Socio Economic Database for Latin America and the Caribbean (SEDLAC), the Gini coefficient was extracted and merged to the GSHS at survey time or closer to the survey time and recoded into tertiles with the highest tertile as reference category.

**Covariables**

Covariables in the GSHS datasets included age, psychosocial factors (bullying, having close friends, feeling lonely and parental support), lifestyle behaviours (cigarette smoking, alcohol consumption) and food insecurity (used as a proxy measure of socio-economic circumstances)

**Moderators**

Sex, a key determinant of overweight/ obesity and suicidal ideation with planning was used to stratify the analyses.

- 1. **Statistical analysis**

**Preliminary descriptive analyses to explore variation across the region.**

a) outcome, exposure, and other covariable frequency distributions for each of the 21 LAC countries.

b) Sample characteristics were stratified by sex.

c) Creation of scatterplots using complete cases to explore between country variation in overweight/obesity or suicide ideation with planning by country-level socioeconomic indicators for boys and girls.

**Initial statistical methods**

STATA 15 was used in all analyses and survey weights were applied using the SVY command.

**Addressing missing data**

Multiple imputation chained equations (MICE). Thirty (30) multiple imputed datasets were generated. Due to absent data, Anguilla and Antigua and Barbuda were not included in the analysis of overweight/obesity, and Chile and Barbados in the analysis on suicidal ideation with planning. Imputed values on the outcomes of interest were dropped and all estimates were combined by using Rubin’s rules.

**Predictive probabilities**

Sex-specific predictive probabilities of overweight/obesity and suicidal ideation with planning were calculated in each country using age-adjusted logistic regression and adjusted for clustering within countries.

**Logistic regressions**

A p-value of less than 0.05 was statistically significant in random effects models. Random effects modelling considers the hierarchical structure of the data by clustering students’ responses (Level 1) within participating countries (Level 2).

We declared the hierarchical nature of the GSHS data with the xtset command with student responses in the survey nested within countries.

**Modelling strategy**

Model 1: Sex-specific random-effects logistic regression models were used to estimate odd ratios (OR) and 95% confidence intervals (CI) for the associations between national indices (HDI, GDP/capita, Gini index) and overweight/obesity and suicidal ideation with planning unadjusted.

Model 2: Model 1 + adjusted for individual risk factors.

Intraclass Correlation Coefficients represents the percentage of between country differences in overweight/obesity and suicidal ideation with planning after adjustment for GDP/HDI/Gini Index (Model 1) and individual risk factors (Model 2).

**For the association between overweight/obesity and suicidal ideation with planning**

Random-effects logistic regression models adjusted for age, psychosocial factors (bullying, having close friends, feeling lonely and parental support), lifestyle behaviours (cigarette smoking, alcohol consumption) and food insecurity

**🡪 Modifications carried out in the revised version**

- 1. **Sensitivity analyses**

Random effects linear regression models were applied using a standardised continuous BMI score (zBMI score) to correct for positive skewness of BMI as well as continuous macro-economic indicators of development and economic inequality to minimise both Type I and Type II errors and increase the precision of estimates.

To explore whether macro-economic indicators were associated with a higher or lower risk of underweight and overweight/obesity compared to normal category of BMI, we applied multinomial logistic regression, adjusted for the non-independence of observations within countries using the Huber-White variance estimator.
