# Supplementary material for: Association of macro-level determinants with adolescent overweight and suicidal ideation with planning: A cross-sectional study of 21 Latin American and Caribbean Countries
Source: PLoS Med. 2020 Dec 29;17(12):e1003443. doi: 10.1371/journal.pmed.1003443 (PMC7771665; doi:10.1371/journal.pmed.1003443)
Supplement: S1 Table — (DOCX) [file pmed.1003443.s003.docx]

*S1 Table: Distribution (%) of analysis’s variables by country*

|  | Argentina | Anguilla | Antigua | Bahamas | Barbados | Belize | Bolivia | B.Virgin Islands | Chile | Costa Rica | Dominica | El Salvador | Guatemala | Guyana | Honduras | Jamaica | Peru | St Kitts & Nevis | Suriname | Trinidad & Tobago | Uruguay |
| --- | --- | --- | --- | --- | --- | --- | --- | --- | --- | --- | --- | --- | --- | --- | --- | --- | --- | --- | --- | --- | --- |
| Survey year | 2012 | 2009 | 2009 | 2013 | 2011 | 2011 | 2012 | 2009 | 2013 | 2009 | 2009 | 2013 | 2009 | 2010 | 2010 | 2012 | 2010 | 2011 | 2009 | 2011 | 2012 |
| N (%) | 27,988 | 943 | 1,244 | 1,350 | 1,625 | 2,102 | 3,535 | 1,655 | 2,033 | 2,669 | 1,638 | 1,883 | 5,511 | 2,362 | 1,595 | 1,748 | 2,864 | 1,722 | 1,692 | 2,782 | 3,488 |
| ***Outcomes*** |  |  |  |  |  |  |  |  |  |  |  |  |  |  |  |  |  |  |  |  |  |
| Underweight | 2.2 | N/A | N/A | 2.5 | 4.7 | 2 | 1.3 | 2.8 | 0.5 | 2.0 | 3.3 | 1.8 | 1.0 | 7.5 | 2.0 | 2.4 | 0.7 | 3.6 | 7.0 | 6.8 | **2.0** |
| Normal weight | 70.0 | N/A | N/A | 52.0 | 63.4 | 61.8 | 76.2 | 57.1 | 56.9 | 70.0 | 70.4 | 69.5 | 71.6 | 76.9 | 76.4 | 78.9 | 79.5 | 63.4 | 73.2 | 63.7 | **71.4** |
| Overweight/obese | 27.8 | N/A | N/A | 45.5 | 31.9 | 36.2 | 22.5 | 40.1 | 42.7 | 27.9 | 26.3 | 28.8 | 27.4 | 15.6 | 21.6 | 18.7 | 19.8 | 32.5 | 19.8 | 29.5 | 26.6 |
| Boys/Girls | 33.7/21 | N/A | N/A | 42.6/48.7 | 31.2/32.3 | 34.5/38.1 | 19.8/26.3 | 39.9/39.7 | 41.2/44.2 | 27.5/27.6 | 24/29.6 | 29.6/28.2 | 28.7/31 | 14.1/17.8 | 19.5/26.1 | 17.4/19.3 | 20/18.3 | 33.1/31.8 | 19.7/20.0 | 32.9/32.6 | 28.6/25.1 |
| Suicide ideation +plan | 12.1 | 12.2 | 13.5 | 12.3 | N/A | 10.8 | 13.7 | 10.9 | N/A | 5.5 | 15.5 | 9.6 | 9.3 | 18.1 | 17.8 | 15.0 | 14.0 | 11.2 | 9.7 | 12.2 | 7.7 |
| Boys/Girls | 6.4/14.8 | 5.2/15.4 | 7.7/17.6 | 6.7/14.6 | N/A | 6.5/11.9 | 7.8/16.3 | 5.8/12.4 | N/A | 3.7/6.4 | 8.8/16.0 | 5.9/12.3 | 5.5/13.4 | 8.5/19.4 | 8.2/17 | 7.2/17.2 | 6.6/18.5 | 7.2/12.0 | 3.8/13.2 | 8.0/12.2 | 3.8/9.2 |
| ***Covariates*** |  |  |  |  |  |  |  |  |  |  |  |  |  |  |  |  |  |  |  |  |  |
| ***Socio-demographic factors*** | |  |  |  |  |  |  |  |  |  |  |  |  |  |  |  |  |  |  |  |  |
| *Age* |  |  |  |  |  |  |  |  |  |  |  |  |  |  |  |  |  |  |  |  |  |
| <12 years old | 2.5 | 13.6 | 3.3 | 18.2 | 2.2 | 23.7 | 4.9 | 20.6 | 10.3 | 1.9 | 19.4 | 2.8 | 4.7 | 1.6 | 2.1 | 15.2 | 2 | 1.2 | 5.7 | 24.4 | 0.7 |
| 13 years old | 17.7 | 19.5 | 31.8 | 38.2 | 22 | 18.4 | 19.7 | 17 | 22.6 | 28.3 | 21.1 | 18.6 | 21.6 | 21.3 | 12.7 | 24.3 | 17.9 | 21.2 | 14.1 | 23 | 20.5 |
| 14 years old | 29.1 | 22.2 | 33.5 | 29.9 | 38.8 | 21 | 28.2 | 20.9 | 20.1 | 28.3 | 20.4 | 35.3 | 29.7 | 29.8 | 31.9 | 26.5 | 29.6 | 33.8 | 20.6 | 23.9 | 31 |
| 15 years old | 27.5 | 19.3 | 30 | 11.1 | 30 | 19.1 | 27.7 | 17.6 | 13.3 | 26.7 | 21.2 | 29.4 | 26.4 | 31.0 | 29.9 | 20.1 | 33.3 | 29.2 | 22.1 | 18.9 | 30.7 |
| > 16 years old | 23.2 | 25.3 | 1.4 | 2.7 | 7.1 | 17.8 | 19.6 | 23.8 | 33.7 | 14.8 | 17.9 | 13.9 | 17.5 | 16.3 | 23.4 | 13.8 | 17.2 | 14.6 | 37.5 | 9.8 | 17.3 |
| *Gender* |  |  |  |  |  |  |  |  |  |  |  |  |  |  |  |  |  |  |  |  |  |
| Boys | 48 | 47 | 47 | 46 | 45 | 47 | 51 | 45 | 50 | 48 | 44.0 | 54 | 46 | 44.0 | 49.0 | 48 | 49 | 44 | 51 | 55 | 46 |
| Girls | 52 | 53 | 53 | 54 | 55 | 53 | 49 | 55 | 50 | 52 | 56.0 | 46 | 54 | 56.0 | 51.0 | 52 | 51 | 56 | 49 | 45 | 54 |
| ***Psychosocial factors*** | |  |  |  |  |  |  |  |  |  |  |  |  |  |  |  |  |  |  |  |  |
| *Loneliness* |  |  |  |  |  |  |  |  |  |  |  |  |  |  |  |  |  |  |  |  |  |
| Never | 67.0 | 68.5 | 57.5 | 66.7 | 61.9 | 54.3 | 64.7 | 62.9 | N/A | 71.0 | 60.1 | 71.5 | 63.4 | 42.4 | 41.6 | 68.7 | 60.0 | 54.9 | 53.8 | 68.0 | 73.3 |
| Sometimes | 23.0 | 21.6 | 30.6 | 23.3 | 27.3 | 32.7 | 24.5 | 26.6 | N/A | 22.6 | 27.0 | 20 | 27.2 | 40.4 | 39.5 | 20.6 | 29.8 | 33 | 31.1 | 22.1 | 19.4 |
| Always | 10.0 | 9.6 | 13.5 | 11.2 | 11.3 | 13.2 | 11.1 | 10.9 | N/A | 6.6 | 13.2 | 9.5 | 10.7 | 12.2 | 14.8 | 10.5 | 10.7 | 12.2 | 14.8 | 10.5 | 7 |
| *Close friends* |  |  |  |  |  |  |  |  |  |  |  |  |  |  |  |  |  |  |  |  |  |
| 3 or more | 72 | 67.9 | 67.1 | 60.1 | 67.4 | 60.4 | 64.3 | 66 | N/A | 72.7 | 60.7 | 73.8 | 73.2 | 50.7 | 59.2 | 73 | 66 | 56.9 | 45.9 | 59.8 | 75.7 |
| 1 or 2 | 22.6 | 23.1 | 24.4 | 30.4 | 26.8 | 31.9 | 27.4 | 25.4 | N/A | 21.6 | 29.8 | 21 | 21 | 38.8 | 30.9 | 20.2 | 28.6 | 31.6 | 36.9 | 31 | 21.3 |
| None | 5.3 | 9 | 8.4 | 9.5 | 5.9 | 7.7 | 8.3 | 8.6 | N/A | 5.7 | 9.5 | 5.2 | 5.8 | 10.5 | 9.9 | 6.7 | 5.4 | 11.5 | 17.2 | 9.2 | 3.1 |
| *Parental understanding* | |  |  |  |  |  |  |  |  |  |  |  |  |  |  |  |  |  |  |  |  |
| Always | 15.7 | N/A | 20.3 | 19.8 | 12.4 | 20.9 | 8.7 | 15.6 | N/A | 6.6 | N/A | 12.1 | N/A | N/A | 24.3 | 9.4 | 7.8 | 14.6 | 16.3 | 22.3 | N/A |
| Sometimes | 16.7 | N/A | 20.2 | 17 | 16 | 18.1 | 12.7 | 16.2 | N/A | 11.8 | N/A | 13 | N/A | N/A | 21.5 | 9.5 | 12.4 | 23.2 | 15.1 | 19.5 | N/A |
| Rarely | 67.6 | N/A | 59.5 | 63.2 | 71.6 | 61 | 78.6 | 68.2 | N/A | 81.6 | N/A | 74.9 | N/A | N/A | 54.1 | 81.2 | 79.8 | 62.2 | 68.6 | 58.2 | N/A |
| *Times bullied past last 30 days* | |  |  |  |  |  |  |  |  |  |  |  |  |  |  |  |  |  |  |  |  |
| 0 days | 75 | 74 | 72.4 | 76.9 | 87.4 | 69.7 | 67.6 | 81.6 | 86.2 | 80.5 | 73.6 | 77.1 | N/A | 62.7 | 62.8 | 69.1 | 52.5 | 78 | 72.8 | 83.3 | 81.4 |
| 1 or 2 days | 18.9 | 16.9 | 19.1 | 16.5 | 9.6 | 22.6 | 27.5 | 13.5 | 11.6 | 16.3 | 18.3 | 18.8 | N/A | 27.3 | 27.7 | 26.1 | 40.6 | 14.4 | 19.4 | 21.1 | 15.7 |
| 3 or more | 6.1 | 9.1 | 8.5 | 6.6 | 3 | 7.6 | 4.8 | 4.9 | 2.2 | 3.1 | 8.2 | 4.1 | N/A | 10.0 | 9.5 | 4.8 | 6.9 | 7.6 | 7.8 | 4.6 | 2.9 |
| *Times attacked thepast 12 months* | |  |  |  |  |  |  |  |  |  |  |  |  |  |  |  |  |  |  |  |  |
| None | 75.7 | 72.1 | 59.9 | 69.9 | 70.8 | 72 | 65 | 69.4 | 80.6 | 86.2 | 65.0 | 80.8 | N/A | 61.0 | 59.3 | 79.5 | 62.1 | 66.1 | 75.2 | 64.6 | 84.1 |
| 1 time | 17.7 | 17.4 | 23.8 | 20.1 | 19.4 | 18 | 26.3 | 20 | 14.7 | 10 | 22.7 | 14.5 | N/A | 24.7 | 26.4 | 16 | 29.9 | 22.1 | 16.8 | 22.6 | 12.4 |
| 2 or more times | 6.6 | 10.5 | 16.3 | 10.1 | 9.8 | 10.1 | 8.8 | 10.6 | 4.7 | 3.8 | 12.4 | 4.7 | N/A | 14.3 | 14.3 | 4.5 | 7.9 | 11.8 | 8 | 12.8 | 3.5 |
| ***Lifestyle factors*** |  |  |  |  |  |  |  |  |  |  |  |  |  |  |  |  |  |  |  |  |  |
| *Smoking days the past 30 days* | |  |  |  |  |  |  |  |  |  |  |  |  |  |  |  |  |  |  |  |  |
| 0 days | 76.4 | 95.1 | 92.5 | 95.0 | 90.6 | N/A | 85.0 | 94.6 | 74.9 | 88.7 | N/A | N/A | N/A | 87.0 | 80.4 | 87.3 | 81.0 | N/A | 87.8 | 90.4 | 87.2 |
| 1 or 2 days | 8.9 | 2.6 | 4.5 | 2.6 | 5.2 | N/A | 9.9 | 3.4 | 9.6 | 5.7 | N/A | N/A | N/A | 7.3 | 9.9 | 7.7 | 13.3 | N/A | 6.4 | 5.2 | 5.2 |
| 3 or more | 14.7 | 2.3 | 3.1 | 2.4 | 4.2 | N/A | 5.1 | 2.0 | 15.5 | 5.7 | N/A | N/A | N/A | 5.8 | 9.7 | 5.0 | 5.6 | N/A | 5.8 | 4.4 | 7.6 |
| *Alcohol consumption the past 30 days* | |  |  |  |  |  |  |  |  |  |  |  |  |  |  |  |  |  |  |  |  |
| 0 days | 46.6 | 55.3 | 55.6 | 72.4 | 52.3 | 70.7 | 81.7 | 65.0 | 68.0 | 73.7 | 48.5 | 81.0 | 81.4 | 59.0 | 46.5 | 84.0 | 70.1 | N/A | 62.3 | 65.0 | 50.8 |
| 1 or 2 days | 26.1 | 25.8 | 24.7 | 16.7 | 24.8 | 17.7 | 12.8 | 21.9 | 16.8 | 16.6 | 29.7 | 7.0 | 11.7 | 26.2 | 27.4 | 10.8 | 21.7 | N/A | 25.1 | 19.7 | 25.6 |
| 3 or more | 27.3 | 18.9 | 19.7 | 10.9 | 23.0 | 11.6 | 5.5 | 13.0 | 15.2 | 9.7 | 21.8 | 12.0 | 6.8 | 14.8 | 26.1 | 5.2 | 8.2 | N/A | 12.6 | 15.4 | 23.6 |
| ***Food security*** |  |  |  |  |  |  |  |  |  |  |  |  |  |  |  |  |  |  |  |  |  |
| *Times gone to bed hungry* | |  |  |  |  |  |  |  |  |  |  |  |  |  |  |  |  |  |  |  |  |
| Never | 62.8 | 58.1 | 56.4 | 51.7 | 58.1 | 62.7 | 37.2 | 57.1 | 72.2 | 79.7 | 65.0 | 65.0 | 65.4 | 53.2 | 43.5 | 64.1 | 47.9 | 59.0 | 61.4 | 51.2 | 76.4 |
| Sometimes | 33.3 | 35.0 | 36.5 | 41.2 | 37.2 | 30.4 | 53.7 | 36.4 | 26.1 | 18.8 | 29.4 | 31.8 | 32.4 | 39.4 | 46.0 | 32.0 | 49.1 | 36.3 | 28.3 | 41.0 | 22.1 |
| Always | 3.8 | 6.9 | 7.1 | 7.0 | 4.7 | 6.9 | 9.0 | 6.5 | 1.7 | 1.5 | 5.6 | 3.2 | 2.2 | 7.4 | 10.5 | 3.9 | 3.1 | 4.7 | 10.4 | 7.8 | 1.5 |
