# Supplementary material for: Association of macro-level determinants with adolescent overweight and suicidal ideation with planning: A cross-sectional study of 21 Latin American and Caribbean Countries
Source: PLoS Med. 2020 Dec 29;17(12):e1003443. doi: 10.1371/journal.pmed.1003443 (PMC7771665; doi:10.1371/journal.pmed.1003443)
Supplement: S2 Table — (DOCX) [file pmed.1003443.s004.docx]

*S2 Table: Girls - Association between national indices of development, income inequality and overweight/obesity, adjusted for individual risk factors*

|  | Model 1 | Model 2 | Model 1 | Model 2 | Model 1 | Model 2 |
| --- | --- | --- | --- | --- | --- | --- |
|  | OR (95% CI)  (p-value) | OR (95% CI)  (p-value) | OR (95% CI)  (p-value) | OR (95% CI)  (p-value) | OR (95% CI)  (p-value) | OR (95% CI)  (p-value) |
| Higher HDI tertile (ref.) |  |  |  |  |  |  |
| Middle HDI tertile | 0.58 (0.40, 0.99)  (0.047) | 0.60 (0.36, 1.00)  (0.048) |  |  |  |  |
| Lower HDI tertile | 0.49 (0.29, 0.82)  (0.007) | 0.51 (0.31-0.84) |  |  |  |  |
| Higher GDP tertile (ref.) |  |  |  |  |  |  |
| Middle GDP tertile |  |  | 0.57 (0.39, 0.84)  (0.004) | 0.58 (0.41, 0.83)  (0.003) |  |  |
| Lower GDP tertile |  |  | 0.59 (0.43, 0.81)  (0.001) | 0.60 (0.45, 0.81)  (0.001) |  |  |
| Higher Gini tertile (ref) |  |  |  |  |  |  |
| Middle Gini tertile |  |  |  |  | 1.32 (0.71, 2.46)  (0.272) | 1.28 (0.70, 2.33)  (0.430) |
| Lower Gini tertile |  |  |  |  | 1.13 (0.59, 2.17)  (0.718) | 1.07 (0.57, 2.03)  (0.830) |
| Age (ref. <=12) |  |  |  |  |  |  |
| 13 |  | 1.11 (0.87, 1.43)  (0.405) |  | 1.05 (0.89, 1.23)  (0.575) |  | 0.86 (0.75, 0.99)  (0.031) |
| 14 |  | 0.98 (0.77, 1.24)  (0.849) |  | 1.14 (0.98, 1.33)  (0.100) |  | 0.73 (0.63, 0.83)  (<0.001) |
| 15 |  | 0.83 (0.66, 1.05)  (0.128) |  | 1.19 (1.02, 1.39)  (0.029) |  | 0.66 (0.57, 0.76)  (<0.001) |
| 16 |  | 0.75 (0.59, 0.95)  (0.016) |  | 1.48 (1.26, 1.74)  (<0.001) |  | 0.67 (0.58, 0.77)  (<0.001) |
| Loneliness (ref.never) |  |  |  |  |  |  |
| Rarely /sometimes |  | 0.92 (0.87, 0.97)  (0.001) |  | 0.94 (0.87, 1.02)  (0.129) |  | 0.93 (0.87, 1.00)  (0.038) |
| Most of the time/always |  | 1.08 (1.01, 1.16)  (0.028) |  | 1.12 (1.01, 1.25)  (0.0340) |  | 1.04 (0.95, 1.14)  (0.430) |
| Close friends (ref. 3 or more) |  |  |  |  |  |  |
| 1 or 2 |  | 0.98 (0.93, 1.03)  (0.476) |  | 0.97 (0.89, 1.04)  (0.390) |  | 0.99 (0.93, 1.06)  (0.825) |
| none |  | 0.98 (0.84, 1.07)  (0.655) |  | 0.93 (0.81, 1.07)  (0.320) |  | 0.96 (0.85, 1.08)  (0.496) |
| Bullied (ref.never) |  |  |  |  |  |  |
| 1or 2 days |  | - 1. (0.94, 1.07)   (0.843) |  | 1.06 (0.96, 1.18)  (0.256) |  | 1.04 (0.95, 1.13)  (0.436) |
| 3 days or more |  | 1.07 (0.99, 1.16)  (0.088) |  | 1.18 (1.03, 1.35)  (0.018) |  | 1.11 (1.00, 1.24)  (0.056) |
| Parental Support |  |  |  |  |  |  |
| Sometimes |  | - 1. (0.95, 1.08)   (0.762) |  | 0.93 (0.84, 1.03)  (0.148) |  | 0.97 (0.89, 1.06)  (0.543) |
| Never/ rarely |  | 1.06 (1.01, 1.21)  (0.030) |  | 1.03(0.94, 1.12)  (0.562) |  | 1.00(0.94, 1.08)  (0.923) |
| Smoking days (ref.none) |  |  |  |  |  |  |
| 1 to 5 days |  | 1.08 (0.99, 1.17)  (0.07) |  | 1.14 (0.99, 1.31)  (0.064) |  | 1.00(0.90, 1.13)  (0.884) |
| 6 or more days |  | 1.21 (1.09, 1.33)  (<0.001) |  | 1.31 (0.11, 1.55)  (0.001) |  | 1.07 (0.93, 1.24)  (0.330) |
| Alcohol drinking days (ref.none) |  |  |  |  |  |  |
| 1 or 2 days |  | 0.95 (0.89, 1.01)  (0.078) |  | 1.00(0.92, 1.10)  (0.938) |  | 0.93 (0.86, 1.00)  (0.052) |
| 3 or more days |  | 0.86 (0.80, 0.92)  (<0.001) |  | 0.98 (0.87, 1.10)  (0.702) |  | 0.91 (0.83, 1.00)  (0.050) |
| Physically attacked (ref.never) |  |  |  |  |  |  |
| 1 time |  | - 1. (0.95, 1.10)   (0.582) |  | 0.93 (0.82, 1.05)  (0.22) |  | 0.95 (0.86, 1.05)  (0.334) |
| 2 or more times |  | 0.98 (0.91, 1.06)  (0.620) |  | 0.95 (0.85, 1.06)  (0.383) |  | 0.93 (0.84, 1.03)  (0.170) |
| Food insecurity (ref.never or sometimes) |  |  |  |  |  |  |
| Most of the time/always |  | 1.05 (0.99, 1.11)  (0.111) |  | 0.99 (0.91, 1.08)  (0.817) |  | 1.01(0.94,1.10)  (0.710) |
| *Intraclass Correlation Coefficient* | *3.6%* | *3.4%* | *3.6%* | *4.1%* | 4.5% | 3.9% |

*Note: Model1 refers to the unadjusted association between macroeconomic indicators and overweight/obesity. Model 2 refers to the adjusted association between macroeconomic indicators and overweight/obesity.*
