# Supplementary material for: Association of macro-level determinants with adolescent overweight and suicidal ideation with planning: A cross-sectional study of 21 Latin American and Caribbean Countries
Source: PLoS Med. 2020 Dec 29;17(12):e1003443. doi: 10.1371/journal.pmed.1003443 (PMC7771665; doi:10.1371/journal.pmed.1003443)
Supplement: S3 Table — (DOCX) [file pmed.1003443.s005.docx]

*S3 Table: Boys - Association between national indices of development, income inequality and with overweight/obesity, adjusted for individual risk factors*

|  | Model 1 | Model 2 | Model 1 | Model 2 | Model 1 | Model 2 |
| --- | --- | --- | --- | --- | --- | --- |
|  | OR (95% CI)  (p-value) | OR (95% CI)  (p-value) | OR (95% CI)  (p-value) | OR (95% CI)  (p-value) | OR (95% CI)  (p-value) | OR (95% CI)  (p-value) |
| Higher HDI tertile (ref.) |  |  |  |  |  |  |
| Middle HDI tertile | 0.65 (0.40, 1.06)  (0.083) | 0.67 (0.42, 1.06)  0.087 |  |  |  |  |
| Lower HDI tertile | 0.44 (0.27, 0.70)  (0.001) | 0.47 (0.29, 0.75)  (0.001) |  |  |  |  |
| Higher GDP tertile (ref.) |  |  |  |  |  |  |
| Middle GDP tertile |  |  | 0.64 (0.45, 0.92)  (0.015) | 0.67 (0.48, 0.95)  (0.023) |  |  |
| Lower GDP tertile |  |  | 0.52 (0.38, 0.69)  (<0.001) | 0.54 (0.41, 0.71)  (<0.001) |  |  |
| Higher Gini tertile (ref) |  |  |  |  |  |  |
| Middle Gini tertile |  |  |  |  | 1.28 (0.78, 2.09)  (0.332) | 1.21 (0.75,1.93)  (0.439) |
| Lower Gini tertile |  |  |  |  | 1.18 (0.70, 1.98)  (0.544) | 1.09 (0.66,1.79)  0.747) |
| Age (ref. <=12) |  |  |  |  |  |  |
| 13 |  | 1.05 (0.94, 1.17)  (0.712) |  | 0.86 (0.72, 1.02)  (0.085) |  | 0.83 (0.71,0.96)  (0.011) |
| 14 |  | 0.85 (0.81, 1.36)  (0.185) |  | 0.84 (0.71, 0.99)  (0.044) |  | 0.65 (0.56,0.76)  (<0.001) |
| 15 |  | 0.79 (0.55, 0.89)  (0.004) |  | 0.86 (0.72, 1.01)  (0.071) |  | 0.51 (0.44,0.60)  (<0.001) |
| 16 |  | 0.56 (0.44, 0.72)  (<0.001) |  | 1.10 (0.93, 1.30)  (0.277) |  | 0.50 (0.43,0.58)  (<0.001) |
| Loneliness (ref. never) |  |  |  |  |  |  |
| Rarely /sometimes |  | 1.01(0.95 1.07)  (0.766) |  | 1.06 (0.96, 1.16)  (0.258) |  | 1.01 (0.93,1.09)  (0.889) |
| Most of the time/always |  | 1.03(0.94, 1.13)  (0.471) |  | 1.07 (0.93, 1.24)  (0.320) |  | 0.95 (0.84,1.07)  (0.418) |
| Close friends (ref. 3 or more) |  |  |  |  |  |  |
| 1 or 2 |  | 1.00 (0.94, 1.06)  (0.958) |  | 1.00 (0.91, 1.09)  (0.998) |  | 0.98 (0.92,1.06)  (0.669) |
| none |  | 1.03 (0.95, 1.13)  (0.462) |  | 0.90 (0.77, 1.04)  (0.153) |  | 0.96 (0.86,1.09)  (0.552) |
| Bullied (ref. never) |  |  |  |  |  |  |
| 1 or 2 days |  | 1.02 (0.95, 1.09)  (0.628) |  | 1.09 (0.98, 1.22)  (0.11) |  | 1.00 (0.92,1.09)  (0.918) |
| 3 days or more |  | 1.07 (0.99, 1.16)  (0.112) |  | 1.22 (1.07, 1.39)  (0.003) |  | 1.11 (0.99,1.24)  (0.067) |
| Parental Support |  |  |  |  |  |  |
| Sometimes |  | 1.04 (0.97, 1.11)  (0.265) |  | 0.90 (0.81, 0.99)  (0.037) |  | 0.98 (0.90,1.06)  (0.589) |
| Never/ rarely |  | 1.04 (0.99, 1.10)  (0.132) |  | 0.92 (0.84, 1.00)  (0.054) |  | 0.96 (0.89,1.03)  (0.293) |
| Smoking days (ref. none) |  |  |  |  |  |  |
| 1 to 5 days |  | 1.08 (0.99, 1.17)  (0.075) |  | 1.03 (0.90, 1.17)  (0.696) |  | 1.07 (0.95,1.19)  (0.259) |
| 6 or more days |  | 1.08 (0.98, 1.20)  (0.117) |  | 1.03 (0.88, 1.21)  (0.710) |  | 1.01 (0.89,1.15)  (0.855) |
| Alcohol drinking days (ref.none) |  |  |  |  |  |  |
| 1 or 2 days |  | 0.87 (0.82, 0.93)  (<0.001) |  | 0.94 (0.85, 1.04)  (0.220) |  | 0.91 (0.84,0.98)  (0.019) |
| 3 or more days |  | 0.82 (0.77, 0.88)  (<0.001) |  | 0.94 (0.84, 1.04)  (0.229) |  | 0.87 (0.80,0.95)  (0.002) |
| Physically attacked (ref. never) |  |  |  |  |  |  |
| 1 time |  | 0.94 (0.87, 1.01)  (0.073) |  | 0.90 (0.80, 1.02)  (0.112) |  | 0.92 (0.83,1.01)  (0.068) |
| 2 or more times |  | 1.04 (0.98, 1.11)  (0.23) |  | 1.05 (0.95, 1.16)  (0.330) |  | 1.02 (0.94,1.11)  (0.623) |
| Food insecurity (ref. never or sometimes) | |  |  |  |  |  |
| Most of the time/always |  | 1.10 (1.04, 1.17)  (0.002) |  | 0.95 (0.86,1.04)  (0.266) |  | 1.06 (0.98,1.15)  (0.142) |
| *Intraclass Correlation Coefficient* | *2.7%* | *2.6%* | *2.7%* | *2.7%* | *2.9%* | *2.7%* |

*Note: Model1 refers to the unadjusted association between macroeconomic indicators and overweight/obesity. Model 2 refers to the adjusted association between macroeconomic indicators and overweight/obesity.*
