# Supplementary material for: Association of macro-level determinants with adolescent overweight and suicidal ideation with planning: A cross-sectional study of 21 Latin American and Caribbean Countries
Source: PLoS Med. 2020 Dec 29;17(12):e1003443. doi: 10.1371/journal.pmed.1003443 (PMC7771665; doi:10.1371/journal.pmed.1003443)
Supplement: S4 Table — (DOCX) [file pmed.1003443.s006.docx]

*S4 Table: Girls - Association between national indices of development, income inequality, and suicidal ideation with planning, adjusted for individual risk factors*

|  | Model 1 | Model 2 | Model 1 | Model 2 | Model 1 | Model 2 |  |
| --- | --- | --- | --- | --- | --- | --- | --- |
|  | OR (95% CI)  (p-value) | OR (95% CI)  (p-value) | OR (95% CI)  (p-value) | OR (95% CI)  (p-value) | OR (95% CI)  (p-value) | OR (95% CI)  (p-value) |  |
| Higher HDI tertile (ref.) |  |  |  |  |  |  |  |
| Middle HDI tertile | 0.89 (0.56,1.42)  (0.617) | 0.80 (0.50,1.27)  (0.339) |  |  |  |  |  |
| Lower HDI tertile | 1.13 (0.71,1.79)  (0.604) | 0.89 (0.56,1.41)  (0.615) |  |  |  |  |  |
| Highest GDP tertile (ref.) |  |  |  |  |  |  |  |
| Middle GDP tertile |  |  | 1.16 (0.76,1.76)  (0.499) | 0.92 (0.60,1.39)  (0.681) |  |  |  |
| Lower GDP tertile |  |  | 1.11 (0.82,1.51)  (0.509) | 1.03 (0.76,1.40)  (0.829) |  |  |  |
| Higher Gini tertile |  |  |  |  |  |  |  |
| Middle Gini tertile |  |  |  |  | 1.18 (0.69,2.00)  (0.052) | 1.16 (0.68,2.00)  (0.856) |  |
| Lower Gini tertile |  |  |  |  | 1.24 (0.74,2.08)  (0.413) | 1.31(0.78, 2.22)  (0.309) |  |
| Age (ref. <=12) |  |  |  |  |  |  |  |
| 13 |  | 1.20 (1.08,1.32)  (<0.001) |  | 1.25 (1.03,1.52)  (0.025) |  | 1.16 (0.68,2.00)  (0.586) |  |
| 14 |  | 1.18 (1.06,1.30)  (0.002) |  | 1.44 (1.87,1.73)  (<0.001) |  | 1.40 (1.10,1.77)  (0.309) |  |
| 15 |  | 1.13 (1.01,1.26)  (0.031) |  | 1.41 (1.17,1.71)  (<0.001) |  | 1.40 (1.11,1.78)  (0.005) |  |
| 16 |  | 0.67 (0.16,2.74)  (0.576) |  | 1.36 (1.12,1.65)  (0.002) |  | 1.32 (1.03,1.67)  (0.027) |  |
| Loneliness (ref. never) |  |  |  |  |  |  |  |
| Rarely /sometimes |  | 1.82 (1.68,1.97)  (<0.001) |  | 1.82 (1.52,1.92)  (<0.001) |  | 1.90 (1.73,2.08)  (<0.001) |  |
| Most of the time/always |  | 5.18 (4.72,5.69)  (<0.001) |  | 5.17 (4.17,5.68)  (<0.001) |  | 5.78 (5.21,6.42)  (<0.001) |  |
| Close friends (ref. 3 or more) |  |  |  |  |  |  |  |
| 1 or 2 |  | 1.19 (1.10,1.28)  (<0.001) |  | 1.19(1.10, 1.28)  (<0.001) |  | 1.22 (1.11,1.33)  (<0.001) |  |
| none |  | 1.55 (1.35,1.77)  (<0.001) |  | 1.55 (1.36,1.78)  (<0.001) |  | 1.51 (1.29,1.76)  (<0.001) |  |
| Bullied (ref. never) |  |  |  |  |  |  |  |
| 1 or 2 days |  | 1.47 (1.33,1.61)  (<0.001) |  | 1.48 (1.33,1.63)  (<0.001) |  | 1.50 (1.35,1.66)  (<0.001) |  |
| 3 days or more |  | 2.06 (1.84,2.31)  (<0.001) |  | 2.06 (1.85,2.30)  (<0.001) |  | 2.08 (1.84,2.36)  (<0.001) |  |
| Parental Support |  |  |  |  |  |  |  |
| Sometimes |  | 1.26 (1.13,1.40)  (<0.001) |  | 1.26 (1.12,1.40)  (<0.001) |  | 1.23 (1.09, 1.39)  (<0.001) | |
| Never/ rarely |  | 2.22 (2.03,2.42)  (<0.001) |  | 2.22 (2.03,2.42)  (<0.001) |  | 2.09 (1.89, 2.31)  (<0.001) | |
| Smoking days (ref. none) |  |  |  |  |  |  |  |
| 1 to 5 days |  | 2.04 (1.82,2.29)  (<0.001) |  | 2.06 (1.84,2.31)  (<0.001) |  | 2.11 (1.87, 2.38)  (<0.001) | |
| 6 or more days |  | 2.53 (2.21,2.91)  (<0.001) |  | 2.22 (2.03,2.42)  (<0.001) |  | 2.61 (2.62, 3.01)  (<0.001) | |
| Alcohol drinking days (ref.none) |  |  |  |  |  |  |  |
| 1 or 2 days |  | 1.88 (1.72,2.06)  (<0.001) |  | 1.85 (1.69,2.03)  (<0.001) |  | 1.86 (1.68, 2.06)  (<0.001) | |
| 3 or more days |  | 2.47 (2.23,2.74)  (<0.001) |  | 2.41 (2.17,2.67)  (<0.001) |  | 2.39 (2.13, 2.69)  (<0.001) | |
| Physically attacked (ref. never) |  |  |  |  |  |  |  |
| 1 time |  | 1.41 (1.26,1.58)  (<0.001) |  | 1.42 (1.27,1.58)  (<0.001) |  | 1.42 (1.26, 1.61)  (<0.001) | |
| 2 or more times |  | 2.19 (1.98,2.41)  (<0.001) |  | 2.18 (1.98,2.40)  (<0.001) |  | 2.33 (2.09, 2.60)  (<0.001) | |
| Food insecurity (ref. never or sometimes) |  |  |  |  |  |  |  |
| Most of the time/always |  | 1.03 (0.94,1.13)  (0.079) |  | 1.03 (0.94,1.13)  (0.540) |  | 1.06 (0.96, 1.19)  (0.380) | |
| *Intraclass Correlation Coefficient* | *2.3%* | *2.2%* | *2.6%* | *2.5%* | *2.9%* | *3.0%* |  |

*Note: Model1 refers to the unadjusted association between macroeconomic indicators and suicidal ideation. Model 2 refers to the adjusted association between macroeconomic indicators and suicidal ideation.*
