# Supplementary material for: Association of macro-level determinants with adolescent overweight and suicidal ideation with planning: A cross-sectional study of 21 Latin American and Caribbean Countries
Source: PLoS Med. 2020 Dec 29;17(12):e1003443. doi: 10.1371/journal.pmed.1003443 (PMC7771665; doi:10.1371/journal.pmed.1003443)
Supplement: S5 Table — (DOCX) [file pmed.1003443.s007.docx]

*S5 Table: Boys -* *Association between national indices of development, income inequality, and suicidal ideation with planning, adjusted for individual risk factors*

|  | Model 1 | Model 2 | Model 1 | Model 2 | Model 1 | Model2 |  |
| --- | --- | --- | --- | --- | --- | --- | --- |
|  | OR (95% CI) | OR (95% CI) | OR (95% CI) | OR (95% CI) | OR (95% CI) | OR (95% CI) |  |
| Higher HDI tertile (ref.) |  |  |  |  |  |  |  |
| Middle HDI tertile | 0.97 (0.49,1.93)  (0.93) | 0.93 (0.50,1.72)  (0.684) |  |  |  |  |  |
| Lower HDI tertile | 1.17 (0.60,2.28)  (0.654) | 0.94 (0.73,2.28)  (0.376) |  |  |  |  |  |
| Highest GDP tertile (ref.) |  |  |  |  |  |  |  |
| Middle GDP tertile |  |  | 1.00 (0.75,1.53)  (0.984) | 0.79 (0.58,1.10)  (0.67) |  |  |  |
| Lower GDP tertile |  |  | 1.04 (0.76,1.42)  (0.822) | 0.96 (0.74,1.25)  (0.383) |  |  |  |
| Higher Gini tertile |  |  |  |  |  |  |  |
| Middle Gini tertile |  |  |  |  | 1.30 (0.74,2.27)  (0.362) | 1.00 (0.58,1.72)  (0.886) |  |
| Lower Gini tertile |  |  |  |  | 1.29 (0.75,2.21)  (0.357) | 1,12 (0.66,1.88)  (0.676) |  |
| Age (ref. <=12) |  |  |  |  |  |  |  |
| 13 |  | 0.77 (0.45,1.32)  (0.336) |  | 0.84 (0.66,1.08)  (0.174) |  | 0.89 (0.65,1.22)  (0.473) |  |
| 14 |  | 0.71 (0.43,1.17)  (0.177) |  | 0.93 (0.73,1.18)  (0.574) |  | 0.95 (0.69,1.29)  (0.723) |  |
| 15 |  | 0.79 (0.48,1.29)  (0.344) |  | 0.87 (0.69,1.11)  (0.256) |  | 0.90 (0.66,1.23)  (0.502) |  |
| 16 |  | 0.75 (0.46,1.24)  (0.261) |  | 0.91 (0.72,1.68)  (0.474) |  | 1.00 (0.73,1.36)  (0.978) |  |
| Loneliness (ref. never) |  |  |  |  |  |  |  |
| Rarely /sometimes |  | 1.71 (1.52,1.92)  (<0.001) |  | 1.71 (1.52,1.92)  (<0.001) |  | 1.79 (1.57,2.05)  (<0.001) |  |
| Most of the time/always |  | 4.97 (4.33,5.69)  (<0.001) |  | 4.87 (4.25,5.80)  (<0.001) |  | 5.09 (4.35,5.94)  (<0.001) |  |
| Close friends (ref. 3 or more) |  |  |  |  |  |  |  |
| 1 or 2 |  | 1.37 (1.22,1.65)  (<0.001) |  | 1.36(1.21, 1.52)  (<0.001) |  | 1.33 (1.17,1.51)  (<0.001) |  |
| none |  | 2.02 (1.72,2.38)  (<0.001) |  | 2.01 (1.70,2.36)  (<0.001) |  | 2.26 (1.88,2.71)  (<0.001) |  |
| Bullied (ref. never) |  |  |  |  |  |  |  |
| 1 or 2 days |  | 1.50 (1.31,1.71)  (<0.001) |  | 1.55 (1.35,1.78)  (<0.001) |  | 1.52 (1.31,1.76)  (<0.001) |  |
| 3 days or more |  | 2.02 (1.72,2.38)  (<0.001) |  | 1.98 (1.71,2.30)  (0.001) |  | 1.96 (1.65,2.33)  (<0.001) |  |
| Parental Support |  |  |  |  |  |  |  |
| Sometimes |  | 1.12(0.97, 1.30)  (0.125) |  | 1.18 (1.01,1.37)  (0.037) |  | 1.11 (0.94,1.32)  (0.221) |  |
| Never/ rarely |  | 1.62 (1.44,1.82)  (<0.001) |  | 1.79(1.58, 2.02)  (<0.001) |  | 1.71 (1.50,1.95)  (<0.001) |  |
| Smoking days (ref. none) |  |  |  |  |  |  |  |
| 1 to 5 days |  | 1.74 (1.50,2.03)  (<0.001) |  | 1.99 (1.70,2.34)  (<0.001) |  | 2.00 (1.70,2.34)  (<0.001) |  |
| 6 or more days |  | 2.45(2.07, 2.90)  (<0.001) |  | 2.92 (2.45,3.23)  (<0.001) |  | 2.86 (2.39,3.43)  (<0.001) |  |
| Alcohol drinking days (ref.none) |  |  |  |  |  |  |  |
| 1 or 2 days |  | 1.51 (1.32,1.73)  (<0.001) |  | 1.46 (1.28,1.67)  (<0.001) |  | 1.52 (1.30,1.77)  (<0.001) |  |
| 3 or more days |  | 2.00 (1.73,2.31)  (<0.001) |  | 1.90 (1.65,2.18)  (<0.001) |  | 1.97 (1.69,2.30)  (<0.001) |  |
| Physically attacked (ref. never) |  |  |  |  |  |  |  |
| 1 time |  | 1.33 (1.15,1.54)  (<0.001) |  | 1.34 (1.16,1.55)  (<0.001) |  | 1.26 (1.08,1.49)  (0.004) |  |
| 2 or more times |  | 1.62 (1.43,1.82)  (<0.001) |  | 1.65 (1.46,1.86)  (<0.001) |  | 1.63 (1.42,1.88)  (<0.001) |  |
| Food insecurity (ref. never or sometimes) |  |  |  |  |  |  |  |
| Most of the time/always |  | 1.19 (1.06,1.34)  (<0.001) |  | 1.06 (0.94,1.21)  (0.342) |  | 1.09 (0.94, 1.27)  (0.380) | |
| *Intraclass Correlation Coefficient* | *2.4%* | *1.8%* | *2.4%* | *1.4%* | *3.1%* | *2.8%* |  |

*Note: Model1 refers to the unadjusted association* *between macroeconomic indicators and suicidal ideation. Model 2 refers to the adjusted association between macroeconomic indicators and suicidal ideation.*
