# Supplementary material for: Association of macro-level determinants with adolescent overweight and suicidal ideation with planning: A cross-sectional study of 21 Latin American and Caribbean Countries
Source: PLoS Med. 2020 Dec 29;17(12):e1003443. doi: 10.1371/journal.pmed.1003443 (PMC7771665; doi:10.1371/journal.pmed.1003443)
Supplement: S6 Table — BMI, body mass index. (DOCX) [file pmed.1003443.s008.docx]

*S6 Table:* *Association between continuous national indices of development, income inequality, and z-BMI, adjusted for individual risk factors*

|  | Girls | Boys | Girls | Boys | Girls | Boys |
| --- | --- | --- | --- | --- | --- | --- |
|  | Coef (95% CI)  (p-value) | Coef (95% CI)  (p-value) | Coef (95% CI)  (p-value) | Coef (95% CI)  (p-value) | Coef (95% CI)  (p-value) | Coef (95% CI)  (p-value) |
| HDI (continuous) | 0.01 (0.01, 0.02) (0.024) | 0.02 (0.01, 0.02) (<0.001) |  |  |  |  |
| GDP (continuous) |  |  | 0.02 (0.01, 0.03)  (<0.001) | 0.02 (0.02, 0.03)  (<0.001) |  |  |
| Gini (continuous) |  |  |  |  | 0.03 (0.02, 0.04)  (<0.001) | -0.04 (-0.05, -0.03)  (<0.001) |
| Age (ref. <=12) |  |  |  |  |  |  |
| 13 | 0.13 (0.08, 0.19)  (<0.001) | 0.07(0.01, 0.13) (0.017) | 0.07 (0.04, 0.11)  (<0.001) | -0.03 (-0.07, 0.01)  (0.12) | 0.16 (-0.06, 0.38)  (0.16) | 0.08 (-0.16, 0.32)  (0.514) |
| 14 | 0.26 (0.21, 0.31) (<0.001) | 0.14(0.08, 0.20) (<0.001) | 0.11 (0.08, 0.14)  (<0.001) | 0.03 (-0.01, 0.07)  (0.127) | 0.64 (0.42, 0.85)  (<0.001) | 0.33 (0.10, 0.56)  (0.006) |
| 15 | 0.32 (0.27, 0.38)  (<0.001) | 0.26 (0.21, 0.32) (<0.001) | 0.22 (0.19, 0.26)  (<0.001) | 0.17 (0.12, 0.21)  (<0.001) | 0.81 (0.60, 1.03)  (<0.001) | 0.83 (0.60, 1.07)  (<0.001) |
| 16 | 0.46 (0.41, 0.52) (<0.001) | 0.44 (0.38, 0.50) (<0.001) | 0.41 (0.02, 0.80)  (0.038) | 0.28 (-0.07, 0.63)  (0.116) | 1.38 (1.15, 1.61)  (<0.001) | 1.60 (1.36, 1.85)  (<0.001) |
| Loneliness (ref. never) |  |  |  |  |  |  |
| Rarely /sometimes | 0.01 (-0.01, 0.04) (0.388) | 0.03 (-0.01, 0.06) (0.101) | -0.01 (-0.04, 0.02) (0.547) | 0.02 (-0.01, 0.06)  (0.195) | 0.01 (-0.10, 0.13)  (0.846) | 0.06 (-0.07, 0.20)  (0.355) |
| Most of the time/always | 0.05 (0.01, 0.09) (0.009) | 0.06(0.01, 0.11) (0.018) | 0.04 (0.00, 0.08)  (0.044) | 0.05 (-0.01, 0.11)  (0.065) | 0.14 (-0.02, 0.31)  (0.075) | 0.20 (-0.01, 0.40)  (0.064) |
| Close friends (ref. 3 or more) |  |  |  |  |  |  |
| 1 or 2 | 0.01 (-0.01, 0.04) (0.27) | 0.01 (-0.02, 0.04) (0.439) | 0.02 (-0.01, 0.05)  (0.023) | 0.09 (-0.02, 0.04)  (0.589) | 0.04 (-0.07, 0.15)  (0.498) | -0.02 (-0.14, 0.11)  (0.815) |
| none | -0.02 (-0.07, 0.03) (0.41) | -0.04 (-0.09, 0.1) (0.065) | -0.05 (-0.10, 0.01)  (0.078) | -0.04 (-0.10, 0.01)  (0.108) | -0.17 (-0.37, 0.04)  (0.113) | -0.26 (-0.47, -0.05)  (0.017) |
| Bullied (ref. never) |  |  |  |  |  |  |
| 1 or 2 days | 0.02 (-0.02, 0.05) (0.835) | 0.01 (-0.03, 0.05) (0.583) | 0.03 (-0.01, 0.06)  (0.136) | 0.02 (-0.03, 0.06)  (0.463) | - 0.12 (-0.26, 0.03)  (0.119) | -0.10 (-0.26, 0.05)  (0.188) |
| 3 days or more | 0.04 (-0.01, 0.09) (0.063) | 0.06 (0.02, 0.11) (0.008) | 0.07 (0.02, 0.12)  (0.007) | 0.09 (0.04, 0.13)  (0.001) | 0.02 (-0.17, 0.21)  (0.826) | 0.15 (-0.04, 0.34)  (0.124) |
| Parental Support |  |  |  |  |  |  |
| Sometimes | -0.03 (-0.06, 0.01) (0.081) | -0.04 (-0.08, -0.01)  (0.035) | -0.04 (-0.08, -0.01)  (0.055) | -0.04 (-0.08,0.01)  (0.023) | -0.06 (-0.20, 0.09)  (0.445) | -0.13 (-0.28, 0.02)  (0.087) |
| Never/ rarely | 0.01 (-0.02, 0.04) (0.646) | -0.02 (-0.05, 0.01) (0.147) | -0.01(-0.04, 0.03)  (0.748) | -0.03 (-0.06,0.01)  (0.087) | 0.16 (0.04, 0.28)  (0.010) | -0.06 (-0.18, 0.07)  (0.937) |
| Smoking days (ref. none) |  |  |  |  |  |  |
| 1 to 5 days | 0.07 (0.02, 0.12) (0.004) | -0.01 (-0.06, 0.04) (0.731) | 0.06 (0.01, 0.11)  (0.021) | -0.01 (-0.05,0.05)  (0.936) | 0.22 (0.02, 0.42)  (0.034) | -0.07 (-0.26, 0.12)  (0.456) |
| 6 or more days | 0.14 (0.08, 0.19) (<0.001) | 0.01 (-0.05, 0.07) (0.778) | 0.12 (0.06, 0.19)  (<0.001) | 0.01 (-0.05, 0.07)  (0.689) | 0.47 (0.22, 0.73)  (<0.001) | 0.08 (-0.16, 0.32)  (0.498) |
| Alcohol drinking days (ref.none) | |  |  |  |  |  |
| 1 or 2 days | 0.03 (-0.01, 0.06) (0.057) | 0.02 (-0.01, 0.05) (0.229) | 0.01 (-0.03, 0.04)  (0.768) | -0.01 (-0.04,0.03)  (0.807) | 0.09 (-0.05, 0.22)  (0.203) | 0.04 (-0.11, 0.18)  (0.611) |
| 3 or more days | 0.02 (-0.02, 0.06) (0.247) | 0.04 (0.01, 0.08)  (0.043) | -0.01 (-0.05, 0.03)  (0.627) | -0.01 (-0.05,0.03)  (0.726) | 0.08 (-0.08, 0.24)  (0.339) | 0.16 (0.01, 0.32)  (0.039) |
| Physically attacked (ref. never) | |  |  |  |  |  |
| 1 time | -0.01 (-0.04, 0.04) (0.953) | -0.02(-0.06, 0.02)  (0.226) | 0.01 (-0.04, 0.04)  (0.964) | -0.04 (-0.09,0.01)  (0.051) | 0.01 (-0.16, 0.19)  (0.88) | -0.13 (-0.29, 0.04)  (0.129) |
| 2 or more times | 0.02 (-0.02, 0.05) (0.433) | 0.01 (-0.03, 0.04)  (0.746) | -0.01 (-0.05, 0.03)  (0.718) | 0.01 (-0.03, 0.05)  (0.694) | 0.14 (-0.03, 0.31)  (0.105) | 0.04 (-0.11, 0.19)  (0.621) |
| Food insecurity (ref. never or sometimes) | |  |  |  |  |  |
| Most of the time/always | 0.01 (-0.02, 0.04)  (0.26) | -0.03 (-0.06, 0.01)  (0.06) | 0.02 (-0.01, 0.05)  (0.246) | -0.01 (-0.05,0.02)  (0.477) | 0.11 (-0.02, 0.24)  (0.093) | -0.21 (-0.35, -0.08)  (0.002) |
| Intraclass Correlation Coefficient | 1.0% | 1.0% | 1.0% | 1.0% | 4.1% | 4.2% |
