# Supplementary material for: Association of macro-level determinants with adolescent overweight and suicidal ideation with planning: A cross-sectional study of 21 Latin American and Caribbean Countries
Source: PLoS Med. 2020 Dec 29;17(12):e1003443. doi: 10.1371/journal.pmed.1003443 (PMC7771665; doi:10.1371/journal.pmed.1003443)
Supplement: S7 Table — (DOCX) [file pmed.1003443.s009.docx]

*S7 Table:* *Association between continuous national indices, income inequality and underweight and overweight/obesity relative to normal weight, adjusted for individual risk factors*

|  |  | | | Girls | | | Boys | | Girls | | | Boys | Girls | Boys |
| --- | --- | --- | --- | --- | --- | --- | --- | --- | --- | --- | --- | --- | --- | --- |
|  |  | | | RR (95% CI)  (p-value) | | | RR (95% CI)  (p-value) | | RR (95% CI)  (p-value) | | | RR (95% CI)  (p-value) | RR (95% CI)  (p-value) | RR (95% CI)  (p-value) |
| Underweight | GDP | | | 1.02 (0.99,1.04)  (0.224) | | | 1.00 (0.98, 1.02)  (0.955) | |  | | |  |  |  |
|  |  | | |  | | |  | |  | | |  |  |  |
|  | HDI | | |  | | |  | | 1.01 (0.98,1.03)  (0.597) | | | 0.99 (0.97,1.01)  (0.931) |  |  |
|  | Gini | | |  | | |  | |  | | |  | 0.98 (0.95,1.02)  (0.358) | 1.02 (0.98,1.05)  (0.342 |
|  | Age (ref. <=12) | | |  | | |  | |  | | |  |  |  |
|  | 13 | | | 0.71 (0.62,0.81)  (<0.001) | | | 0.71 (0.06, 0.87)  (<0.001) | | 0.70 (0.61, 0.79)  (<0.001) | | | 0.71 (0.58, 0.86)  (<0.001) | 0.68 (0.58, 0.80)  (<0.001) | 0.73 (0.59, 0.89)  (0.002) |
|  | 14 | | | 0.49 (0.42,0.56)  (<0.001) | | | 0.50 (0.41, 0.62)  (<0.001) | | 0.47 (0.042, 0.54)  (<0.001) | | | 0.49 (0.40, 0.60)  (<0.001) | 0.46 (0.39, 0.54)  (<0.001) | 0.51 (0.41, 0.64)  (<0.001) |
|  | 15 | | | 0.40 (0.33,0.49)  (<0.001) | | | 0.32 (0.27, 0.37)  (<0.001) | | 0.39 (0.32, 0.47)  (<0.001) | | | 0.31 (0.26, 0.37)  (<0.001) | 0.38 (0.30, 0.48)  (<0.001) | 0.32 (0.26, 0.40)  (<0.001_ |
|  | 16 | | | 0.31 (0.26,0.38)  (<0.001) | | | 0.20 (0.16, 0.25)  (<0.001) | | 0.30 (0.25, 0.36)  (<0.001) | | | 0.20 (0.16, 0.25)  (<0.001) | 0.30 (0.24, 0.37)  (<0.001) | 0.21 (0.16, 026)  (<0.001) |
|  | Loneliness (ref. never) | | |  | | |  | |  | | |  |  |  |
|  | Rarely /sometimes | | | 0.93 (0.84,1.02)  (<0.001) | | | 0.99 (0.90, 1.10)  (0.911) | | 0.92 (0.84, 1.01)  (0.094) | | | 0.98 (0.89, 1.07)  (0.605) | 0.93 (0.85, 1.03)  (0.161) | 0.98 (0.88, 1.09)  (0.752) |
|  | Most of the time/always | | | 0.96 (0.85,1.08)  (0.466) | | | 0.99 (0.86, 1.14)  (0.914) | | 0.95 (0.84, 1.07)  (0.404) | | | 0.98 (0.86, 1.12)  (0.788) | 0.97 (0.86, 1.08)  (0.554) | 0.99 (0.85, 1.14)  (0.838) |
|  | Close friends (ref. 3 or more) | | | | | |  | |  | | |  |  |  |
|  | 1 or 2 | | | 1.09 (0.96,1.24)  (0.172) | | | 1.01 (0.91, 1.12)  (0.859) | | 1.10 (0.97, 1.24)  (0.136) | | | 1.01 (0.91, 1.12)  (0.851) | 1.09 (0.96, 1.24)  (0.16) | 1.02 (0.92, 1.14)  (0.691) |
|  | none | | | 1.04 (0.93,1.16)  (0.527) | | | 1.14 (0.98, 1.32)  (0.08) | | 1.04 (0.92, 1.17)  (0.538) | | | 1.14 (0.99, 1.32)  (0.076) | 1.04 (0.93, 1.15)  (0.528) | 1.16 (0.99, 1.35)  (0.059) |
|  | Bullied (ref. never) | | |  | | |  | |  | | |  |  |  |
|  | 1 or 2 days | | | 0.97 (0.85,1.10)  (0.647) | | | 0.97 (0.89, 1.06)  (0.532) | | 0.95 (0.83, 1.10)  (0.517) | | | 0.97 (0.88, 1.06)  0.479) | 0.96 (0.82, 1.11)  (0.55) | 0.98 (0.89, 1.08)  (0.759) |
|  | 3 days or more | | | 1.07 (0.95,1.20)  0.264 | | | 1.02 (0.93, 1.13)  (0.619) | | 1.06 (0.93, 1.19)  (0.383) | | | 1.01 (0.91, 1.12)  (0.847) | 1.06 (0.94, 1.20)  (0.368) | 1.04 (0.93, 1.16)  (0.543) |
|  | Parental Support | | |  | | |  | |  | | |  |  |  |
|  | Sometimes | | | 1.04 (0.96,1.13)  (0.312) | | | 1.08 (0.99, 1.19)  (0.098) | | 1.05 (0.97, 1.14)  (0.238) | | | 1.08 (0.98, 1.19)  (0.103) | 1.04 (0.96, 1.14)  (0.338) | 1.06 (0.97, 1.16)  (0.219) |
|  | Never/ rarely | | | 0.97 (0.89,1.06)  (0.56) | | | 1.04 (0.94, 1.15)  (0.483) | | 0.98 (0.90, 1.06)  (0.605) | | | 1.03 (0.93, 1.15)  (0.526) | 0.95 (0.86, 1.06)  (0.365) | 1.00 (0.90, 1.12)  (0.952) |
|  | Smoking days (ref. none) | | |  | | |  | |  | | |  |  |  |
|  | 1 to 5 days | | | 0.78 (0.66,0.93)  (0.007) | | | 0.98 (0.83, 1.17)  (0.828) | | 0.78 (0.66, 0.92)  (0.003) | | | 0.98 (0.82, 1.16)  (0.798) | 0.78 (0.66, 0.91)  (0.002) | 0.97 (0.81, 1.16)  (0.726) |
|  | 6 or more days | | | 0.70 (0.53,0.93)  (0.013) | | | 0.88 (0.71, 1.10)  (0.266) | | 0.69 (0.52, 0.91)  (0.009) | | | 0.89 (0.73, 1.08)  (0.236) | 0.69 (0.53, 0.91)  (0.009) | 0.87 (0.72, 1.06)  (0.157) |
|  | Alcohol drinking days (ref.none) | | | | | |  | |  | | |  |  |  |
|  | 1 or 2 days | | | 0.97 (0.83,1.14)  (0.721) | | | 0.90 (0.81, 0.99)  (0.037) | | 0.99 (0.83, 1.17)  (0.873) | | | 0.91 (0.81, 1.03)  (0.124) | 0.97 (0.81, 1.16)  (0.739) | 0.92 (0.83, 1.03)  (0.141) |
|  | 3 or more days | | | 0.94 (0.80,1.11)  (0.464) | | | 0.79 (0.69, 0.91)  (0.001) | | 0.96 (0.81, 1.14)  (0.634) | | | 0.81 (0.70, 0.94)  (0.006) | 0.94 (0.79, 1.12)  (0.496) | 0.83 (0.73, 0.94)  (0.003) |
|  | Physically attacked (ref. never) | | | | | |  | |  | | |  |  |  |
|  | 1 time | | | 0.98 (0.85,1.13)  (0.804) | | | 0.93 (0.85, 1.02)  (0.108) | | 0.99 (0.85, 1.14)  (0.846) | | | 0.92 (0.84, 1.01)  (0.093) | 0.97 (0.84, 1.13)  (0.73) | 0.93 (0.85, 1.02)  (0.144) |
|  | 2 or more times | | | 0.82 (0.70,1.02)  (0.126) | | | 1.08 (0.97, 1.19)  (0.157) | | 0.92 (0.82, 1.02)  (0.120) | | | 1.07 (0.97, 1.18)  (0.165) | 0.91 (0.82, 1.01)  (0.078) | 1.07 (0.97, 1.18)  (0.184) |
|  |  |  |  | |  |  | |  | |  |  |  |  |  |
|  | Most of the time/always | | | 1.12 (1.02,1.22)  (0.021) | | | 1.21 (1.12, 1.32)  (<0.001) | | 1.13 (1.02, 1.24)  (0.014) | | | 1.20 (1.10, 1.31)  (<0.001) | 1.12 (1.02, 1.23)  (0.018) | 1.21 (1.12, 1.31)  (<0.001) |
| Overweight/Obesity | GDP (continuous) | | | 1.03 (1.01,1.06)  (0.003) | | | 1.04 (1.02,1.05)  (<0.001) | |  | | |  |  |  |
|  | HDI (cont) | | |  | | |  | | 1.01 (0.98, 1.04)  (0.599) | | | 1.02 (1.00, 1.04)  (0.011) |  |  |
|  | Gini (cont) | | |  | | |  | |  | | |  | 1.01  (0.98, 1.05)  (0.436) | 0.99 (0.97, 1.01)  (0.245) |
|  | Age (ref. <=12) | | |  | | |  | |  | | |  |  |  |
|  | 13 | | | 0.83 (0.70,0.99)  (0.033) | | | 0.80 (0.59,1.10)  (0.175) | | 0.78 (0.65, 0.93)  (0.007) | | | 0.74 (0.53, 1.03)  (0.078) | 0.77 (0.64, 0.93)  (0.005) | 0.71 (0.52, 0.96)  (0.028) |
|  | 14 | | | 0.85 (0.71,1.01)  (0.064) | | | 0.77 (0.58,1.02)  (0.071) | | 0.78 (0.62, 0.98)  (0.03) | | | 0.70 (0.52, 0.95)  (0.021) | 0.78 (0.61, 0.98)  (0.035) | 0.66 (0.49, 0.89)  (0.007) |
|  | 15 | | | 0.84 (0.70,1.05)  (0.123) | | | 0.77 (0.57,1.04)  (0.091) | | 0.76 (0.58, 1.00)  (0.046) | | | 0.69 (0.50, 0.96)  (0.027) | 0.76 (0.57, 1.00)  (0.046) | 0.65 (0.46, 0.91)  (0.013) |
|  | 16 | | | 1.01 (0.82,1.26)  (0.908) | | | 0.97 (0.71,1.33)  (0.864) | | 0.93 (0.71, 1.22)  (0.583) | | | 0.87 (0.62, 1.21)  (0.404) | 0.92 (0.70, 1.22)  (0.583) | 0.83 (0.59, 1.17)  (0.279) |
|  | Loneliness (ref. never) | | |  | | |  | |  | | |  |  |  |
|  | Rarely /sometimes | | | 0.98 (0.92,1.05)  (0.621) | | | 1.08 (1.01,1.16)  (0.032) | | 0.97 (0.90, 1.05)  (0.47) | | | 1.07 (1.01, 1.15)  (0.031) | 0.95 (0.88, 1.03)  (0.198) | 1.04 (0.96, 1.12)  (0.327) |
|  | Most of the time/always | | | 1.15 (0.99,1.35)  (0.075) | | | 1.10 (0.97,1.25)  (0.137) | | 1.13 (0.98, 1.32)  (0.099) | | | 1.11 (0.97, 1.27)  (0.127) | 1.10 (0.92, 1.31)  (0.28) | 1.09 (0.95, 1.25)  (0.226) |
|  | Close friends (ref. 3 or more) | | | | | |  | |  | | |  |  |  |
|  | 1 or 2 | | | 1.02 (0.91,1.14)  (0.759) | | | 0.99 (0.91,1.08)  (0.89) | | 1.02 (0.92, 1.14)  (0.644) | | | 1.00 (0.91, 1.10)  (0.981) | 1.01 (0.91, 1.13)  (0.803) | 0.99 (0.90, 1.09)  (0.796) |
|  | none | | | 0.99 (0.82,1.19)  0.911) | | | 0.92 (0.76,1.11)  (0.383) | | 0.98 (0.83, 1.16)  (0.839) | | | 0.94 (0.78, 1,14)  (0.532) | 0.96 (0.80, 1.16)  (0.697) | 0.92 (0.77, 1.10)  (0.348) |
|  | Bullied (ref. never) | | |  | | |  | |  | | |  |  |  |
|  | 1 or 2 days | | | 0.95 (0.83,1.09)  (0.435) | | | 1.02 (0.92,1.14)  (0.70) | | 0.91 (0.77, 1.07)  (0.26) | | | 0.98 (0.87, 1.11)  (0.791) | 0.90 (0.76, 1.07)  (0.227) | 0.96 (0.84, 1.09)  (0.211) |
|  | 3 days or more | | | 1.08 (0.94,1.24)  (0.26) | | | 1.17 (1.06,1.29)  (0.001) | | 1.04 (0.91, 1.20)  (0.552) | | | 1.13 (1.02, 1.26)  (0.022) | 1.03 (0.89, 1.19)  (0.722) | 1.11 (0.98, 1.26)  (0.256) |
|  | Parental Support | | |  | | |  | |  | | |  |  |  |
|  | Sometimes | | | 0.98 (0.87,1.26)  (0.719) | | | 0.90 (0.82,0.99)  (0.026) | | 0.99 (0.89, 1.11)  (0.889) | | | 0.92 (0.82, 1.03)  (0.132) | 0.99 (0.89, 1.10)  (0.846) | 0.93 (0.83, 1.04)  (0.211) |
|  | Never/ rarely | | | 1.10 (0.90,1.34)  (0.096) | | | 0.93 (0.85,1.00)  (0.063) | | 1.11 (0.98, 1.25)  (0.095) | | | 0.94 (0.85, 1.03)  (0.161) | 1.10 (1.00, 1.22)  (0.04) | 0.95 (0.87, 1.04)  (0.256) |
|  | Smoking days (ref. none) | | |  | | |  | |  | | |  |  |  |
|  | 1 to 5 days | | | 1.05 (0.87,1.26)  (0.629) | | | 1.00 (0.83,1.20)  (0.983) | | 1.02 (0.84, 1.24)  (0.846) | | | 0.97 (0.79, 1.18)  (0.766) | 1.02 (0.83, 1.24)  (0.87) | 0.96 (0.78, 1.19)  (0.733) |
|  | 6 or more days | | | 1.10 (0.90,1.34)  (0.37) | | | 1.02 (0.87,1.21)  (0.776) | | 1.09 (0.86, 1.37)  (0.486) | | | 0.98 (0.83, 1.16)  0.791) | 1.13 (0.90, 1.42)  (0.307) | 0.98 (0.83, 1.16)  (0.833) |
|  | Alcohol drinking days (ref.none) | | | | | |  | |  | | |  |  |  |
|  | 1 or 2 days | | | 0.93 (0.79,1.08)  (0.325) | | | 0.90 (0.83,0.99)  (0.032) | | 0.97 (0.84, 1.24)  (0.664) | | | 0.92 (0.82, 1.02)  (0.105) | 1.03 (0.88, 1.19)  (0.737) | 0.93 (0.83, 1.05)  (0.261) |
|  | 3 or more days | | | 0.88 (0.75,1.04)  (0.126) | | | 0.92 (0.80, 1.05)  (0.092) | | 0.94 (0.80, 1.09)  (0.389) | | | 0.93 (0.80, 1.08)  (0.364) | 1.02 (0.86, 1.21)  (0.818) | 0.97 (0.82, 1.14)  (0.691) |
|  | Physically attacked (ref. never) | | | | | |  | |  | | |  |  |  |
|  | 1 time | | | 0.93 (0.81,1.06)  (0.283) | | | 0.88 (0.79,0.98)  (0.016) | | 0.93 (0.81, 1.06)  (0.273) | | | 0.89 (0.81, 0.97)  (0.011) | 0.92 (0.80, 1.05)  (0.226) | 0.89 (0.81, 0.98)  (0.015) |
|  | 2 or more times | | | 1.02 (0.91,1.15)  (0.729) | | | 1.06 (0.96,1.18)  (0.266) | | 1.03 (0.91, 1.15)  (0.660) | | | 1.09 (0.99, 1.22)  (0.091) | 1.01 (0.90, 1.14)  (0.866) | 1.10 (0.99, 1.22)  (0.082) |
|  | Food insecurity (ref. never or sometimes) | | |  | | |  | |  | | |  |  |  |
|  |  |  |  |  |  |  |  |  |  |  |  |  |  |  |
|  | Most of the time/always | | | 1.07 (0.97,1.18)  (0.172) | | | 0.94 (0.81,1.09)  (0.442) | | 1.08 (0.97, 1.21)  (0.168) | | | 0.96 (0.83, 1.11)  (0.606) | 1.07 (0.97, 1.19)  (0.164) | 0.94 (0.81, 1.10)  (0.439) |
