# Supplementary figures and images for: Association of macro-level determinants with adolescent overweight and suicidal ideation with planning: A cross-sectional study of 21 Latin American and Caribbean Countries
Source: PLoS Med. 2020 Dec 29;17(12):e1003443. doi: 10.1371/journal.pmed.1003443 (PMC7771665; doi:10.1371/journal.pmed.1003443)

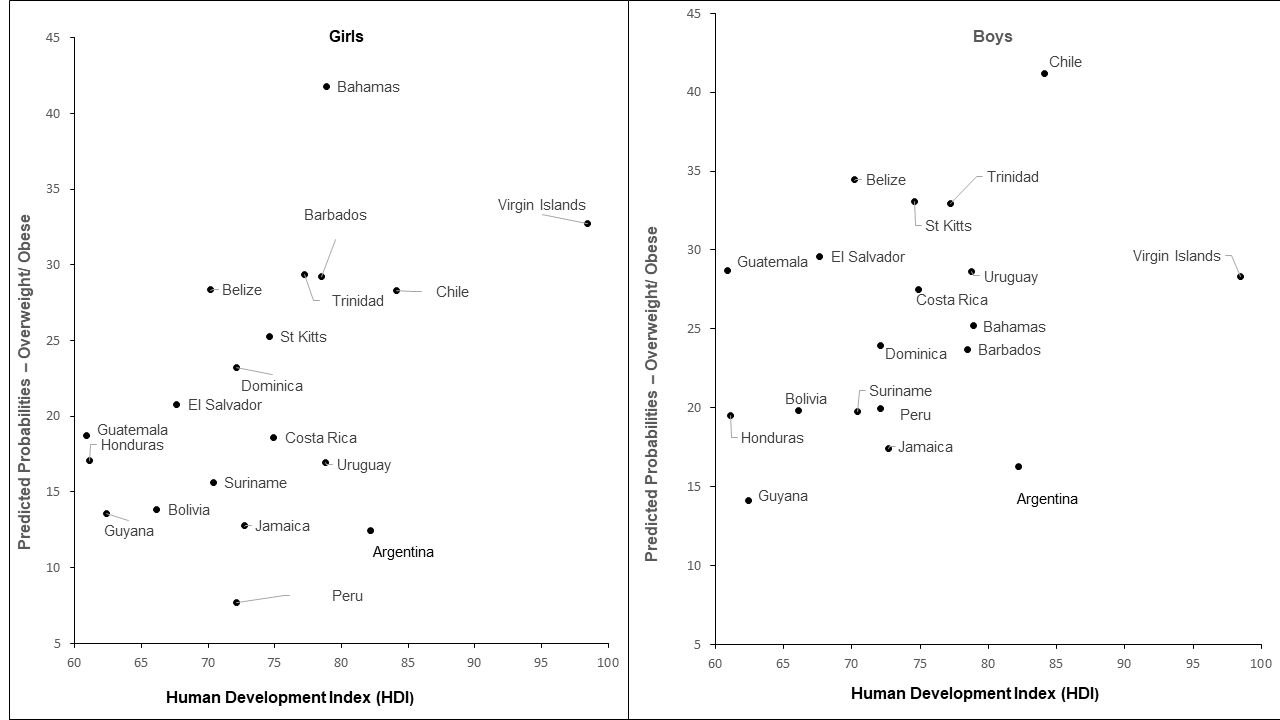

Supplement: S1 Fig — HDI, human development index. (TIFF) [file pmed.1003443.s010.tiff]

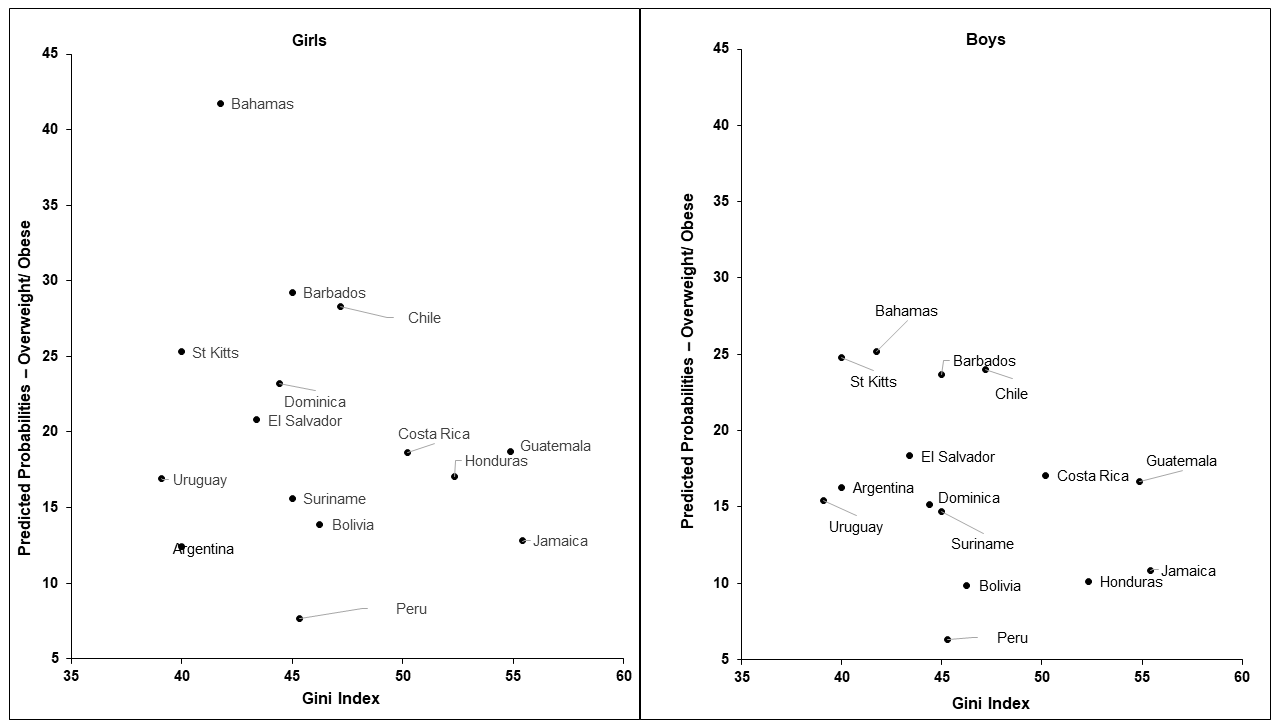

Supplement: S2 Fig — (TIFF) [file pmed.1003443.s011.tiff]

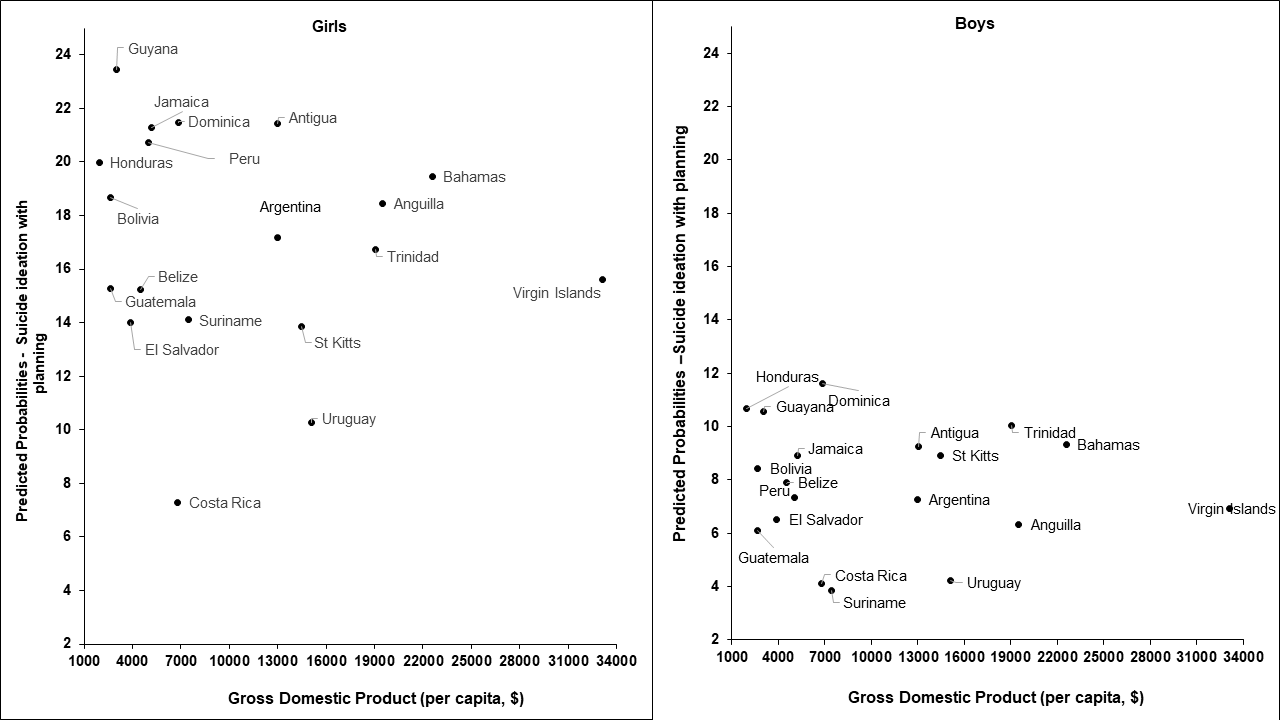

Supplement: S3 Fig — GDP, gross domestic product. (TIFF) [file pmed.1003443.s012.tiff]

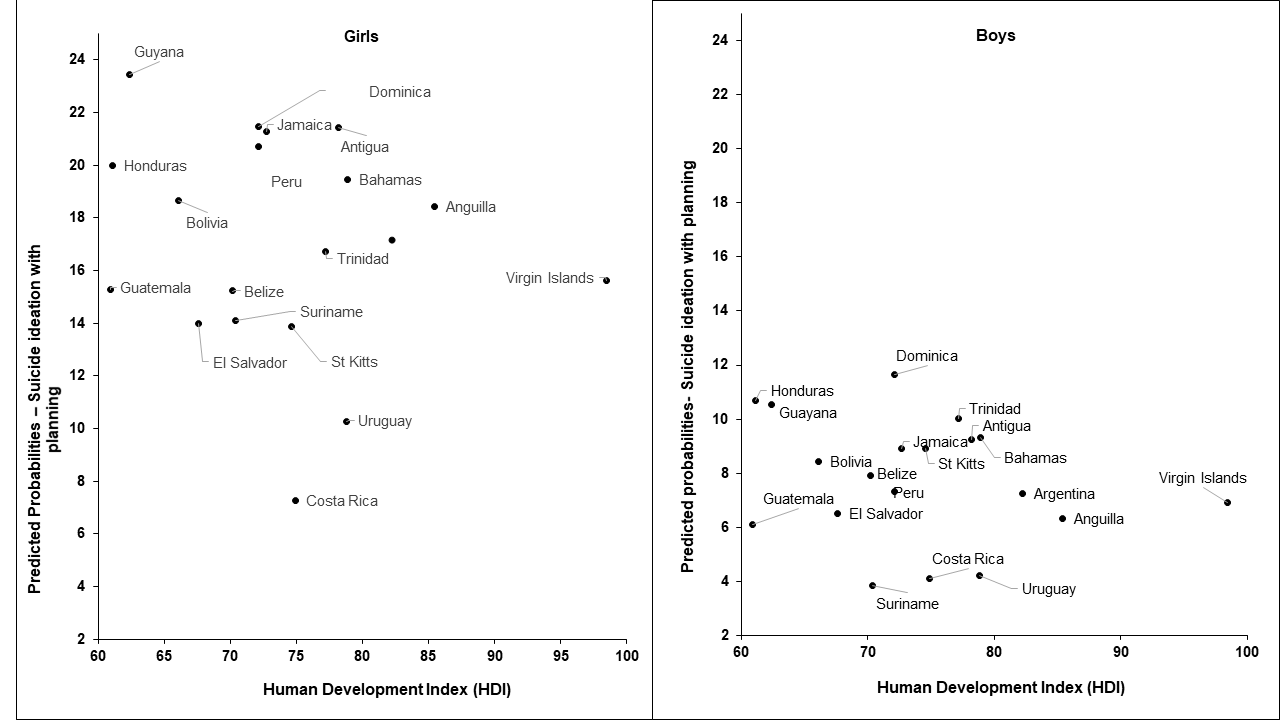

Supplement: S4 Fig — HDI, human development index. (TIFF) [file pmed.1003443.s013.tiff]

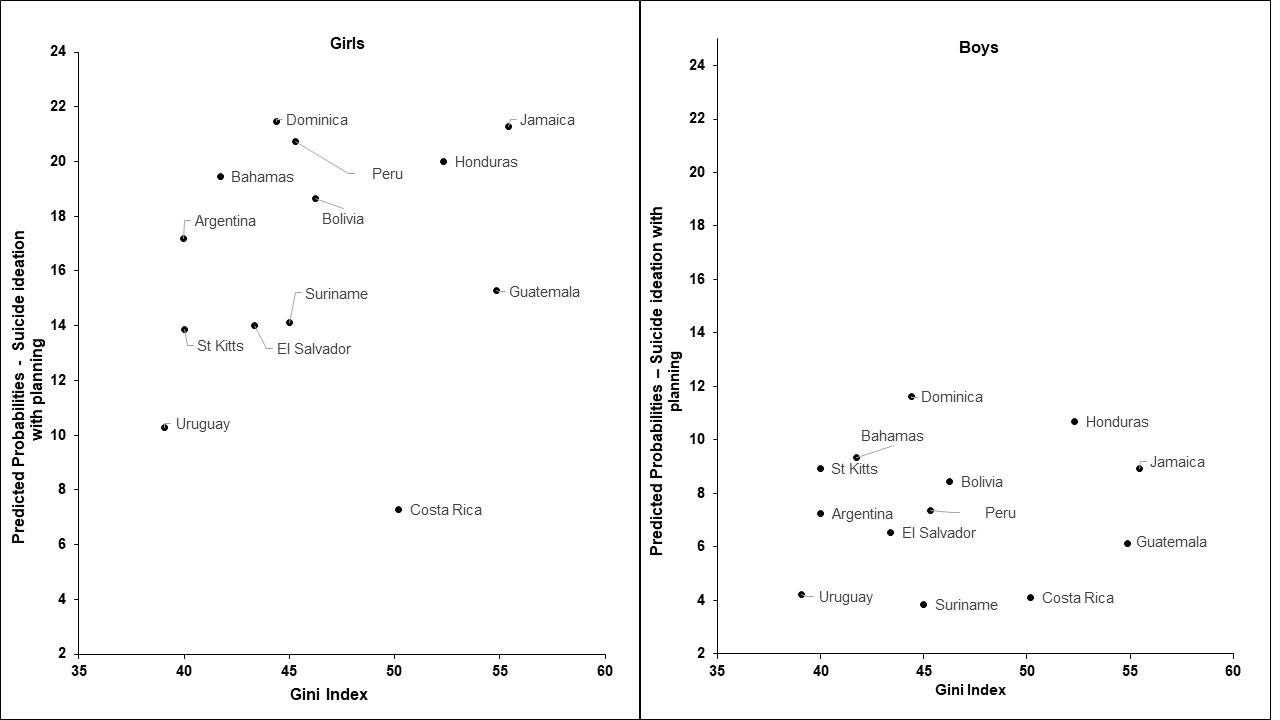

Supplement: S5 Fig — (TIFF) [file pmed.1003443.s014.tiff]
