# Supplementary material for: Association of macro-level determinants with adolescent overweight and suicidal ideation with planning: A cross-sectional study of 21 Latin American and Caribbean Countries
Source: PLoS Med. 2020 Dec 29;17(12):e1003443. doi: 10.1371/journal.pmed.1003443 (PMC7771665; doi:10.1371/journal.pmed.1003443)
Supplement: Attachment — Submitted filename: Reviewers_response_Final 8thSeptember .docx [file pmed.1003443.s015.docx]

**Editorial comments**

Abstract
Please provide p values as needed
Please define LAC on first view
Limitations of your work should be included in a sentence at the end of the methods and findings section

Author summary
At this stage, we ask that you include a short, non-technical Author Summary of your research to make findings accessible to a wide audience that includes both scientists and non-scientists. The Author Summary should immediately follow the Abstract in your revised manuscript. This text is subject to editorial change and should be distinct from the scientific abstract. Please see our author guidelines for more information: [https://eur03.safelinks.protection.outlook.com/?url=https%3A%2F%2Fjournals.plos.org%2Fplosmedicine%2Fs%2Frevising-your-manuscript%23loc-author-summary&amp;data=01%7C01%7Cseeromanie.harding%40kcl.ac.uk%7Ca496fa67c8784d24e53908d82d6a56e4%7C8370cf1416f34c16b83c724071654356%7C0&amp;sdata=pTASql3CqLMqATkuPjaUNz43lxY%2BpPd%2FAqFzmO7fEv4%3D&amp;reserved=0](https://eur03.safelinks.protection.outlook.com/?url=https%3A%2F%2Fjournals.plos.org%2Fplosmedicine%2Fs%2Frevising-your-manuscript%23loc-author-summary&data=01%7C01%7Cchristelle.elia%40kcl.ac.uk%7C82cdf700f9a64818507908d82d6dc4d6%7C8370cf1416f34c16b83c724071654356%7C0&sdata=fR41eYHYhaE5oj2SzjyF6HZ9xVYz6Z1CsF0sqbSEHvE%3D&reserved=0)
Throughout-please use square brackets for references and Vancouver style for bibliography

**R: We have now implemented the suggested changes in the revised manuscript.**

Introduction
On page 5 please revise “prevalence of undernutrition is highest in Guyana and income Trinidad with levels as high 19-20%”

**R: Thank you for the suggestion. We have now revised the sentence.**

Did your study have a prospective protocol or analysis plan? Please state this (either way) early in the Methods section.

a) If a prospective analysis plan (from your funding proposal, IRB or other ethics committee submission, study protocol, or other planning document written before analyzing the data) was used in designing the study, please include the relevant prospectively written document with your revised manuscript as a Supporting Information file to be published alongside your study, and cite it in the Methods section. A legend for this file should be included at the end of your manuscript. 

b) If no such document exists, please make sure that the Methods section transparently describes when analyses were planned, and when/why any data-driven changes to analyses took place. 

c) In either case, changes in the analysis-- including those made in response to peer review comments-- should be identified as such in the Methods section of the paper, with rationale.
Methods
Please introduce GSHS, LMICs on first view

**R: All suggestion are now addressed in the revised manuscript.**

Results
Please include p values throughout, providing exact p values unless p<0.001

Please include the Discussion sub heading on Page 15
Please temper all assertions of primacy by adding “to our knowledge” or similar 
Page 15 please introduce SSB on first view
Page 16 could you use an alternative to “better off countries”
Please introduce NCDs on first view
Please ensure that the study is reported according to the STROBE guideline, and include the completed checklist as Supporting Information. When completing the checklist, please use section and paragraph numbers, rather than page numbers. Please add the following statement, or similar, to the Methods: "This study is reported as per the Strengthening the Reporting of Observational Studies in Epidemiology (STROBE) guideline (S1 Checklist).
Please report your study according to the relevant guideline, which can be found here: [https://eur03.safelinks.protection.outlook.com/?url=http%3A%2F%2Fwww.equator-network.org%2F&amp;data=01%7C01%7Cseeromanie.harding%40kcl.ac.uk%7Ca496fa67c8784d24e53908d82d6a56e4%7C8370cf1416f34c16b83c724071654356%7C0&amp;sdata=qip9taWl0MIGR10QxgQhb4NTb9nC2czHlXF1PBSaPDE%3D&amp;reserved=0](https://eur03.safelinks.protection.outlook.com/?url=http%3A%2F%2Fwww.equator-network.org%2F&data=01%7C01%7Cchristelle.elia%40kcl.ac.uk%7C82cdf700f9a64818507908d82d6dc4d6%7C8370cf1416f34c16b83c724071654356%7C0&sdata=0VAEzdV6pBzW2rRzv6nZ8AjjZbScqK%2BEOD%2F3LLbm2lg%3D&reserved=0)
The Data Availability Statement (DAS) requires revision. For each data source used in your study: 

a) If the data are freely or publicly available, note this and state the location of the data: within the paper, in Supporting Information files, or in a public repository (include the DOI or accession number).

b) If the data are owned by a third party but freely available upon request, please note this and state the owner of the data set and contact information for data requests (web or email address). Note that a study author cannot be the contact person for the data.

c) If the data are not freely available, please describe briefly the ethical, legal, or contractual restriction that prevents you from sharing it. Please also include an appropriate contact (web or email address) for inquiries (again, this cannot be a study author).

**R: We have now addressed the above comments in the revised manuscript.**

**Comments from the reviewers:

Reviewer #1:**

I confine my remarks to statistical aspects of this paper. The general approach is fine but I have some issues to resolve before I can recommend publication.

In the introduction, the authors use causal language where it is not appropriate, e.g. on the bottom of page 5. The relation between teen behaviors and parental relationships are complex and probably involve cause in both directions as well as from exogenous variables.

**R: We have now rephrased our sentences to avoid the use of causal language in the text.**

On p. 5 "fourth leading cause" is basically meaningless. It depends on how you label the causes. Give a rate (suicides per thousand per year or whatever),

**R: We have amended this sentence to say: *“Worldwide, suicide is one the leading causes of death in youth which accounts for an estimated 6% of all deaths among young people “***

p 6  I don't understand why Argentina was excluded. Having a large population is not a reason for exclusion. If you want to analyze data country by country, that's fine, but meta-analytic type methods are designed to deal with different sized samples.

**R: We agree with the Reviewer and we have included Argentina in our analysis. All Tables and Figures were also amended.**
p. 7 Do not divide GDP and HDI into tertiles. This throws away information and increases both type I and type II error, while decreasing the precision of estimates. Leave them continuous and use splines to investigate nonlinearity.

Table 2  BMI is a terrible measure of overweight and dichotomizing it makes it worse.  See e.g. my blog post [https://eur03.safelinks.protection.outlook.com/?url=https%3A%2F%2Fmedium.com%2Fpeter-flom-the-blog%2Fwhy-bmi-is-a-bad-measure-of-obesity-and-what-is-better-f8a62fc9ca49%3Fsource%3Dfriends_link%26sk%3D4b2ea559ab12853beb577764f83d151a&amp;data=01%7C01%7Cseeromanie.harding%40kcl.ac.uk%7Ca496fa67c8784d24e53908d82d6a56e4%7C8370cf1416f34c16b83c724071654356%7C0&amp;sdata=ZDt8Pyl1FhdjttGVlbtnQCnsmmoFCD5VJvQnvR%2FGkws%3D&amp;reserved=0](https://eur03.safelinks.protection.outlook.com/?url=https%3A%2F%2Fmedium.com%2Fpeter-flom-the-blog%2Fwhy-bmi-is-a-bad-measure-of-obesity-and-what-is-better-f8a62fc9ca49%3Fsource%3Dfriends_link%26sk%3D4b2ea559ab12853beb577764f83d151a&data=01%7C01%7Cchristelle.elia%40kcl.ac.uk%7C82cdf700f9a64818507908d82d6dc4d6%7C8370cf1416f34c16b83c724071654356%7C0&sdata=CDNEWRgccj8mqVAK8WLeZBD87ROK50Fx1lojZDu29q8%3D&reserved=0) or many other citations. Maybe you can't get a good measure of obesity, but you could leave BMI continuous. Similarly, use age in years, times attacked and so on. Think about how you want to model the count variables, but treating them using optimal scaling seems reasonable.

**R: We have included a sensitivity analysis section where we have explored all macroeconomic indicators as continuous variables and BMI as a zBMI score. On Page 11 we are now saying that: *“In sensitivity analyses we have applied random effects linear regression by using a standardised continuous BMI score to correct for positive skewness of BMI as well as continuous macro-economic indicators of development and economic inequality to minimise both Type I and Type II errors and increase the precision of estimates. Since there was no evidence of quadratic effects of macroeconomic indicators was observed, so only linear effects were modelled. To explore whether macro-economic indicators were associated with a higher or lower risk of underweight and overweight/obese compared to normal category of BMI ,we applied multinomial logistic regression, adjusted for the non-independence of observations within countries by using the Huber-White variance estimator.”* Further on Page 15 we have included the following text: “*Supplementary Table 6 documents that among boys, a*** ***1000$ per capita increase was associated with a 0.02 (95% CI 0.02 to 0.03, p <0.001) SD in BMI, a unit increase in HDI with a 0.02 (95% CI 0.01 to 0.02, p<0.001) SD increase, while a unit increase in Gini was associated with a 0.04 (95% CI 0.03 to 0.05, p<0.001) SD decrease in BMI, after adjustment for covariates. For girls, 1000$ per capita increase was associated with 0.02 (95% CI 0.01 to 0.03, p<0.001) SD increase in BMI, a unit increase in HDI with a 0.01 (95% CI 0.01 to 0.02, p=0.024) SD increase, while a unit increase in Gini was associated with a 0.03 (95% CI 0.02 to 0.04, p<0.001) SD increase in BMI, after adjustment for covariates. Further, the results of the multinomial logistic regression showed associations only between macro-economic indicators and overweight/obese, but not underweight (See Supplementary Table 7).”***

page 9 Please describe the weighting variable and how it was calculated.

**R: We have added information on the weighting variable which was used in all statistical analyses. On Page 9 we have included the following text: “*The weighting formula used is W=W1*W2*f1*f2*f3, where W1 corresponds to the inverse probability of selecting each school, W2 refers to the inverse probability of selecting each classroom, while f1 refers to a school-level non-response adjustment factor, f2 to a student-level non response adjustment factor calculated by classroom, and f3 to a post-stratification factor calculated by sex within grade.”***

**Reviewer #2:**

Many thanks for inviting me to review this valuable contribution to the literature on the economic determinants of health. This cross-sectional study of an international survey finds increased GDP is associated with a reduced chance of adolescent overweight, but not suicide. The study provides a useful contribution in the Latin American and Caribbean setting. However, I have a few suggestions the authors may wish to consider that might improve their manuscript. 

Major issues
1.    Exclusion of Argentina: It seems somewhat problematic to exclude Argentina from all analyses. I appreciate the authors concern that its inclusion could skew the results but I wonder if at least a sensitivity analysis could be included, given the likely policy interest within Argentina and the relative lack of analyses within Latin America more generally.

**R: Please see our reply to Reviewer 1, second comment. Thank you.**

2.    Choice of macroeconomic exposures: An additional variable that might be worth investigating would be income inequality (e.g. measured through the Gini index - available through the UNU-WIDER database, for example)

***R: We thank the Reviewer for this suggestion. We were able to extract the Gini index and have included in the analyses. Added to pages 8 and 9 ‘Information on income inequality was extracted from the World Inequality Income Database (WIID- https://www4.wider.unu.edu/) which includes data from the Socio-Economic Database for Latin America and the Caribbean (SEDLAC), a harmonized set of 6 indicators based on a collection of surveys. The SEDLAC is considered as one of the two main references for cross-country inequality comparisons in the region. The GINI coefficient was extracted and merged to the GSHS at survey time or closer to survey time at country level. The GINI coefficient measures the inequality among values of a frequency distribution (levels of income). When multiple observations for Gini were available for a country at survey year or a year close to survey time, an average of high-quality observations as judged by WIID was used (28). A Gini coefficient of 0 expresses perfect equality, where all values are the same, while a Gini coefficient of 1 expresses maximal inequality among values (e.g., for a large number of people where only one person has all the income or consumption and all others have none, the Gini coefficient will be nearly 1). For the main analysis , the Gini index was recorded into tertiles, with the high Gini tertile being the reference category.’***

3.    Tertiles for the exposure: While I appreciate that tertiles are easy to interpret, this categorisation does seem slightly arbitrary and might reduce the statistical power for detecting associations. Would a sensitivity analysis treating the exposure as a continuous variable be worth considering?

**R: We have now implemented the suggested analyses as per our reply to Reviewer 1, third comment.**

4.    Comparability of response rates: Some further information on the comparability of response rates across countries would be helpful.

**R: We have now included in Table 1 and added to page 6 ‘ Response rates varied from 60% in Chile to 90% in British Virgin Islands and Trinidad and Tobago.’**

Minor issues
1.    Measurement of body mass index: It wasn't clear to me how BMI was measured and whether there was any validation of the measures?

**R: We have now clarified on Pages 6 and 7 that: “*The GSHS contained variables for underweight, overweight and obesity. The underweight category of BMI was derived using international age- and sex-specific child body mass index (BMI) cut-points (> -2 Standard Deviations from the median for age and sex based on WHO growth reference curves). The overweight/obese category of BMI reflects a BMI cut point of > +1 Standard Deviation from the median for age and sex based on WHO growth reference curves. The main statistical analyses focused on the association between macro-economic indicators and overweight/obesity according to the analysis plan and the small number of underweight participants.”***

2.    "A p-value of less than 0.05 was statistically significant" - I would suggest avoiding this binary approach to assessing associations. See: Sterne JAC, Cox DR, Smith GD. Sifting the evidence—what's wrong with significance tests? Another comment on the role of statistical methods. BMJ (Clinical research ed). 2001;322(7280):226-31.

**R: We agree with the Reviewer comment and refrained from a binary interpretation of p-values.**

3.    Typos: Discussion "Individuals level factors such [as] age". "shifted from a low income to upper middle income [country]". "The higher prevalence [of] suicidal behaviour".

**R: We have now rectified the typos. Thank you**

**Reviewer #3:**

This is an interesting paper, but I have some questions about the methods and suggestions about the presentation. I think one of the challenges is that there is so much included - there are two health outcomes, two measures of economic development that are potential explanatory factors, an interaction with food insecurity (which is listed as a covariate), the relationship between the two outcomes, all presented for males and females. This results in so many tables and graphs that much of the results are in supplementary tables. In light of this, I think the paper needs a rewrite, with a focus on the key research questions throughout, rather than trying to present everything.

**R:** **We hope the revised version is clearer. We have reduced the number of graphs in the text and included only key abridged tables in the text. Full tables are included as supplementary tables.**
 ***1.    The final sentence of the first paragraph is not referenced.***

**R: We have now added three references in the revised manuscript.**

***2.    Other than the fact that they are important outcomes for adolescents and young people, it is not clear enough from the beginning why you have focused on overweight and suicide. Although later in the Introduction you cite studies that show a relationship between them, you acknowledge later that this could be due to confounding. Is there a logical reason for studying these two outcomes together, for example is one on the causal pathway or the other?***

**R: We have amended the text and we stress that there is a possible bi-directional association between overweight/obesity and suicide related behaviour. In addition, we stress that the two variables are likely to be sharing common risk factors pointing towards similar macro-economic determinants. On Page 5, we are now stating:** ***“Overweight, obesity, and suicide-related behaviours are among the most prevalent and debilitating physical and mental conditions affecting young people (19, 20), with the two conditions sharing common risk factors (poverty, mental illness, early life adverse events) which point towards similar macroeconomic determinants (15, 21, 22). Systematic reviews show conflicting evidence of the association between overweight/obesity and suicidal behaviours. In their review of 15 studies, Amiri and Behnezhad reported an inverse association between overweight/obesity with suicide mortality and attempted suicide, but a positive association between overweight/obesity and with suicidal ideation (5). A possible bi-directional association was noted with overweight/obesity being associated with an increased risk of suicidal behaviours, while drugs used to treat severe mental illness were strongly associated with weight gain. Another systematic review by Perera and colleagues showed that underweight was significantly associated with an increased risk of completed suicide and obesity and overweight were significantly associated with a decreased risk of completed suicide relative to normal weight (23). There were only two adolescent studies in these reviews, both of which showed a positive association with suicidal thoughts, one with overweight (24) and the other with obesity (25). “***

3.    The third paragraph of the Introduction is about malnutrition, undernutrition, and excess weight. There is not a corresponding paragraph about suicide behaviour.

**R: We have amended the text and now there is a corresponding paragraph on suicide related behaviours. On Page 5 we are now stating: *“Worldwide, suicide is one the leading causes of death in youth which accounts for an estimated 6% of all deaths among young people.*** ***A recent review of adolescent suicidal behaviours in low- and middle-income countries (LMICs) showed that across the LAC region, physical attacks, bullying, loneliness, lack of parental support, and alcohol and tobacco use were strongly related to suicidal behaviours. Adolescent suicide rates are highest in Guyana (18.3 per 100,000) and lowest in Jamaica (1.2/100,000) (17). Trends in deaths from self-harm and inter-personal violence have been increasing at different rates across the region, between 1990 and 2017, with the highest increase among 15-19-year olds in Latin America . As in high income countries, structural determinants such as economic poverty are associated with suicidal related behaviours. Current alcohol use and lack of peer support are risk factors, while strong parental relationships are important protective factors – reducing the prevalence of serious planning for suicide by up to a third (16, 18)].”***

***4.    The fourth paragraph of the Introduction references papers that assessed the relationship between overweight or BMI and suicide behaviours. Were they looking for a linear association, or did they consider that there may be an increase in risk for both underweight and overweight?***

**R: We have added more information on the cited systematic reviews. Please see Page 5.**

5.    In the sentence after refs 16&17, there is an 'and' missing between alcohol and tobacco.

6.    In the Methods section, there is an 'and' missing on line 6.

**R: We have revised the text accordingly.**

7.    In Table 2, BMI is categorised as 'normal weight' or 'overweight/obese'. This surprised me as, based on the Introduction, I thought you would be investigating underweight as well as overweight (and undernutrition is picked up in the Discussion again, so this seems to be quite an omission).

**R: Initially we focused on the association between macro-economic indicators and overweight/obesity according to the analysis plan and because of the small proportion of participants being underweight. We agree this was an omission and we further conducted multinomial logistic to explore the risk of underweight or overweight/obese compared to normal weight according to macroeconomic indicators. Please see page 15 ‘Associations between macro-economic indicators and underweight were not evident (Supplementary Table 7).’**

**We agree with the reviewer and we now use the term overweight/obese throughout the text. Therefore , we now refer to 'overweight/obesity' throughout the text.**

8.    Given the hierarchical nature of the data (individuals nested within countries) are the models fitted multilevel models? Please give more details, explaining how the methods used are appropriate to this data structure.

**R: We have added the following text on Page 11: *“Random effects modelling considers the hierarchical structure of the data by clustering students’ responses (Level 1) within participating countries (Level 2). This was done in STATA by declaring the hierarchical nature of the GSHS data with the xtset command by declaring that student responses in the survey were nested within countries.”***

9.    In the Statistical Analysis section, you introduce an interaction between the measures of development and food insecurity. This variable has not been mentioned in the Introduction, and a rationale is needed for testing this interaction and not others. The results do not appear in any of the tables - this may be one analysis too many.

**R: We agree with the reviewer’s concerns and we therefore have dropped the interaction from statistical analyses.**

10.    I question the term 'suicidal behaviour' used throughout the paper. My understanding is that suicidal behaviour includes suicide and attempted suicide, and that the term 'suicidal ideation' - defined as thoughts about taking action to end one's life, including: identifying a method, having a plan, and/or having intent to act - is closer to the outcome you are using.

**R: We have now used ‘suicidal ideation’ term throughout.**

11.    Figures 1 and 2 present 'predictive probabilities'. What are these predictions based on, and are they different to the observed rates? Please explain in the Methods section.

**R: We have now clarified in the text on Page 9 that: “Predictive probabilities were calculated using margins command in Stata, which provide estimates of relative risk or risk difference in study outcomes measures associated with each predictor in the context of logistic regression”.**

12.    It is not clear, apart from lack of space, why results for suicidal behaviours are in supplementary tables while those for overweight are in the main paper, this feels a bit like selective reporting.

**R: We accept the reviewer’s concerns and we have now added these results to Table 5 in the main body of the text.**

13.    The fifth line from the bottom of p11, I'm afraid I can't see where 52%-62% comes from in the tables, and 45%-51% in the next line.

**R: We agree with the reviewer and we have now removed these percentages.**

14.    The results presented in the first two paragraphs of p12 are not entirely clear. For example, lines 2/3, frequent bullying and cigarette smoking was associated WITH a higher likelihood of overweight FOR GIRLS. The first two sentences of the second paragraph needs to be carefully checked. I think it says 'overweight' instead of 'suicidal behaviours' and the results do not seem to correspond entirely to those in the table.

**R: The text has been checked and amended.**

15.    The interaction  presented at the end of the first paragraph on p12 is between food insecurity and lower v higher income countries. Is this according to the classifications in Table 1, or according to GDP/HDI as it says in the Statistical Analysis section? These results do not appear in any tables.

**R: We have removed this interaction terms as the reviewer suggested earlier (Please see our reply to comment 9).**

16.    If the section 'Overweight and suicide' is to be included, it follow logically from the aims and methods, so please make sure you explain why this is a useful analysis, and describe it in the 'Statistical Analysis' section. The results do not appear to be presented in a table.

**R: We have made amendments in the introduction and the statistical analysis section on Pages . We have presented the results in the text (Page 15). ‘*Overweight/obesity and suicidal ideation There was a positive association between overweight and suicidal behaviours for girls (OR 1.12, 95% CI 1.02 to 1.24, p=0.009) after adjusting for age, psychosocial factors, health related behaviours and food insecurity. A weak positive association was found for boys (1.09, 95% CI 0.96 to 1.24, p=0.182) after statistical adjustment for covariates.’***

17.     The first half of p15 are 'Main Findings', but after that it reads like the Discussion.

**R: Thank you. We have now eliminated ‘Main Findings’.**

18.    P17, last sentence before 'Strengths and Limitations', should read 'the higher prevalence OF suicidal behaviour..' There is a well-recognised paradox here, that suicide is higher in males than females, which is not acknowledged in your Discussion, but deserves mention.

**R: We agree with the comment of the reviewer and we have now mentioned this gender suicide paradox on Page 18**

19.    In the Stengths and Limitations, you mention that income inequality could not be found for all countries, might be worth adding to this sentence 'so was not included in the analysis'.

**R: We have benefited by Reviewer’s comment pointing us to the WIID and we have now used the Gini index for the analysis. Please see our reply to Reviewer 2, point 2.**

20.    Figure 1. The country labels need to be included in a key. There is a missing footnote. Why is the y axis on the right rather than the left of the graphs.

**R: We have now corrected these graphs. Y axis is on the left of the graphs.**

21.    Table 4. It is not clear why there are 4 columns rather than 2 (Boys and Girls). In places the columns appear to be repeated but this is not the case all the way through. Please give all Intraclass Correlation Coefficients to 1dp. It might be helpful to the reader to say that this is the 'Model 2' referred to in the paper.
22.    Table 5. The row 'rarely/sometimes' has an entry for '192' which should be '1.92'. The row '1 to 5 days' is missing a comma in the first column of numbers.

**R: Thank you for the suggestion. We have addressed the error.**

23.    Supplementary Table 1. The title needs to be rephrased. It looks like you have presented percentages, but this is not stated. Please present all numbers to 1dp. It is not clear what the two numbers presented for M/F are, as they do not sum to 100%.

R: **R: Thank you for the suggestion. We have rephrased the title as suggested.**

24.    Supplementary Table 2. . It might be helpful to the reader to say that this is the 'Model 1' referred to in the paper. Please change the group labels to read 'Higher GDP tertile', 'Middle GDP tertile' and 'Lower GDP tertile'. Similar for HDI, and this applies to Tables 4 & 5.

R: **R: Thank you for the suggestion. We have now implemented the suggested changes.**
