# Supplementary material for: Association of macro-level determinants with adolescent overweight and suicidal ideation with planning: A cross-sectional study of 21 Latin American and Caribbean Countries
Source: PLoS Med. 2020 Dec 29;17(12):e1003443. doi: 10.1371/journal.pmed.1003443 (PMC7771665; doi:10.1371/journal.pmed.1003443)
Supplement: Attachment — Submitted filename: Response to editors and production issues.docx [file pmed.1003443.s016.docx]

**Requests from Editors:**

Please revise your title according to PLOS Medicine's style. Your title must be nondeclarative and not a question. It should begin with main concept if possible. "Effect of" should be used only if causality can be inferred, i.e., for an RCT. Please place the study design ("A randomized controlled trial," "A retrospective study," "A modelling study," etc.) in the subtitle (ie, after a colon).

Abstract-please provide brief participant demographics

Please remove "166 million young people" as most populous countries were not included and this can be misleading

Please remove the sentences about violence in the abstract and provide the background relevant to your current work

Please add to the abstract "sample sizes ranged from X for Anguilla to Y for Argentina", for example 

Please include the dominance of Argentinians in the dataset as a limitation

**R: We have now implemented the suggested changes in the revised title and abstract**.

Abstract conclusions:* Please address the study implications without overreaching what can be concluded from the data; the phrase "In this study, we observed ..." may be useful. * Please interpret the study based on the results presented in the abstract, emphasizing what is new without overstating your conclusions. * Please avoid vague statements such as "these results have major implications for policy/clinical care". Mention only specific implications substantiated by the results. * Please avoid assertions of primacy ("We report for the first time....")

**R: we have now reworded the abstract’s conclusions to address the comment above.**

Please replace all iterations of “obese” with the phrase “with obesity” or “with overweight” throughout, in line with people first language principles

Please replace gender with “sex”

The author summary needs to be in bullet points please, with clear subsections. Please see our author guidelines for more information: https://journals.plos.org/plosmedicine/s/revising-your-manuscript#loc-author-summary

Please include all funding information in the article metadata only, this is not needed in the main text

Please use square brackets for references

I suggest rewording the introduction lines 71-78 to avoid repeating the text provided in the abstract

Please add details of GSHS data in the data availability statement, including a link to the dataset

Discussion- please discuss limitations of your study design, especially the bidirectional relationship between suicide ideation and overweight/obesity that may influence your findings in addition to the usual confounding factors.

**R: We have now made all the necessary changes required in the revised manuscript and the metadata.**

Please use paragraphs and sections in the STROBE checklist, as page numbers are likely to change

Did your study have a prospective protocol or analysis plan? Please state this (either way) early in the Methods section.

a) If a prospective analysis plan (from your funding proposal, IRB or other ethics committee submission, study protocol or other planning document written before analyzing the data) was used in designing the study, please include the relevant prospectively written document with your revised manuscript as a Supporting Information file to be published alongside your study, and cite it in the Methods section. A legend for this file should be included at the end of your manuscript.

b) If no such document exists, please make sure that the Methods section transparently describes when analyses were planned, and when/why any data-driven changes to analyses took place.

c) In either case, changes in the analysis-- including those made in response to peer review comments-- should be identified as such in the Methods section of the paper, with rationale.

Please add full access details for reference 19.

**R: We have now addressed all the above comments in the revised manuscript.**

**Production Issues:**

FIGURES and GRAPHICS:

1. Please provide all main figures in .tif or .eps format. For more information about how to convert your figure files, please see our guidelines:  Once you've converted your files to .tif or .eps, please also make sure that your figures meet our format requirements:

2. Please ensure that all main figures are referenced in the manuscript as Fig 1, Fig 2, etc. (including capitalization), rather than Figure 1, Figure 2, etc. Please note, however, that the file names themselves must not include the space, i.e. Fig1.tif, Fig2.tif. Please ensure that all main figure files are clearly labeled as such in the File Inventory.

METADATA:

1. Please provide a detailed Financial Disclosure statement. This is published with the article, therefore should be completed in full sentences and contain the exact wording you wish to be published.

- Please clarify all sources of funding (financial or material support) for your study. List the grants (with grant number) or organizations (with url) that supported your study, including funding received from your institution.

- State the initials, alongside each funding source, of each author to receive each grant.

- State what role the funders took in the study. If the funders had no role in your study, please state: “The funders had no role in study design, data collection and analysis, decision to publish, or preparation of the manuscript.”

- If any authors received a salary from any of your funders, please state which authors and which funders.

If you did not receive any funding for this study, please state: “The authors received no specific funding for this work.”

2. In accordance with PLOS' data policy, please ensure that your Data Availability Statement in the submission form clearly identifies how readers can access your data. Note that it will be typeset as it is written so please ensure it is in complete sentences and appears as you would like it to in the published version.

ARTICLE FILE:

1. Please indicate the corresponding author on the title page with an asterisk (*) and include the corresponding author's email address. Only an asterisk and the email address itself are required.

2. Please ensure that all author names are spelled correctly in the manuscript file and the submission form, including the use of a middle name/initials, and any special characters (accents, umlauts, etc.). Please place first/given names before last/family names. We noticed that your author names are formatted differently on the submission form and the title page (the title page only includes first initials rather than full names), and ask that you ensure they match verbatim. Please be aware that correct spelling and formatting of author names in the article file is vital for accurate indexing in PubMed.

3. Please ensure that your Ethics Statement is available in the Methods section of your manuscript in its entirety, under the subheading "Ethics Statement".

4. We have noted that you have the following present in your manuscript: Financial Disclosure (Funding)

We will remove these section from your manuscript file as this information is automatically pulled from the submission form. Please make sure that the information in the submission form is correct.

ACKNOWLEDGMENTS:

1. Please remove the funding information from the Acknowledgments section of your manuscript file. The published version is pulled automatically from the “Financial Disclosure” field in our submission form and must not be duplicated. Please therefore take this opportunity to check that the information in this field is correct and completed in full.

REFERENCES:

1. Please make sure all references are cited in ascending numerical order in the text.

SUPPORTING INFORMATION:

1. Please replace the page numbers in your STROBE statement with paragraph numbers per section (e.g. "Methods, paragraph 1") since the page numbers of the final published paper may be different from the page numbers in the current manuscript.

2. Please ensure that each supporting information file has a legend at the end of the manuscript file, after the Reference list.

3. Please note that your submission cannot contain any references to generic "supporting information" files and instead must refer to specific files using our naming convention (S1 Fig, S1 Table, S1 Text, S1 Data, etc.)

Before resubmitting your paper, please ensure that all files referenced in the manuscript are present in the File Inventory, as missing files will result in further delays. Please also ensure that all file names adhere to our naming conventions and that all in-text citations and supporting information file legends/captions do as well.

Please note that once your paper is through to production it is difficult to alter the figures, so this is your best opportunity to ensure that they are correct for publication. It is also important to understand that any changes or additions to data are likely to require a further round of editorial review, which delays publication.

**R: We have now addressed all the suggested changes required in the manuscript and the submission form.**
